# Evaluating the role of *MEN1* gene expression and its clinical significance in breast cancer patients

**DOI:** 10.1371/journal.pone.0288482

**Published:** 2023-07-12

**Authors:** Sheersh Massey, Mohammad Aasif Khan, Safia Obaidur Rab, Saad Mustafa, Asifa Khan, Zoya Malik, Rahimunnisa Shaik, Mohit Kumar Verma, SVS Deo, Syed Akhtar Husain

**Affiliations:** 1 Human Genetics Laboratory, Department of Biosciences, Jamia Millia Islamia, New Delhi, India; 2 Division of Hematology and Medical Oncology, Department of Medicine, University of Texas Health San Science Center at Antonio (UTHSCSA), San Antonio, TX, United States of America; 3 Department of Clinical Laboratory Sciences, College of Applied Medical Sciences, King Khalid University, Abha, Saudi Arabia; 4 Department of Surgical Oncology BRA-IRCH, All India Institute of Medical Sciences (AIIMS), New Delhi, India; University of the Punjab, PAKISTAN

## Abstract

**Background:**

Breast cancer is a multifactorial disease which involves number of molecular factors that are critically involved in proliferation of breast cancer cells. *MEN1* gene that is traditionally known for its germline mutations in neuroendocrine tumors is associated with high risk of developing breast cancer in females with MEN1 syndrome. However, the paradoxical role of *MEN1* is reported in sporadic breast cancer cases. The previous studies indicate the functional significance of *MEN1* in regulating breast cells proliferation but its relevance in development and progression of breast cancer is still not known. Our study targets to find the role of *MEN1* gene aberration and its clinical significance in breast cancer.

**Methods:**

Breast tumor and adjacent normal tissue of 142 sporadic breast cancer patients were collected at the time of surgery. The expression analysis of *MEN1* mRNA and protein was done through RT-PCR, immunohistochemistry and western blotting. Further to find the genetic and epigenetic alterations, automated sequencing and MS-PCR was performed respectively. Correlation between our findings and clinical parameters was determined using appropriate statistical tests.

**Results:**

*MEN1* expression was found to be significantly increased in the breast tumor tissue with its predominant nuclear localization. The elevated expression of *MEN1* mRNA (63.38% cases) and protein (60.56% cases) exhibited a significant association with ER status of the patients. Most of the cases had unmethylated (53.52%) *MEN1* promoter region, which can be a key factor responsible for dysregulated expression of *MEN1* in breast cancer cases. Our findings also revealed the significant association of *MEN1* mRNA overexpression with Age and lymph node status of the patients.

**Conclusion:**

Our results indicate upregulated expression of *MEN1* in sporadic breast cancer patients and it could be critically associated with development and advancement of the disease.

## Introduction

The recent decade has witnessed a tremendous rise in incidence of breast cancer and according to globocan data; it is the 2^nd^ leading cause of death among women globally [1]. Predominantly the breast cancer can originate by hyper-proliferation of milk producing glands or ductal epithelial cells of the breast [2, 3]. The multifarious nature of origin and progression of the disease makes it more challenging for the treatment. Hence, meticulous understanding of breast cancer associated genes as well as their pathogenic mechanism is crucial to develop preventive and therapeutic methods [4, 5].

Multiple Endocrine Neoplasia 1 (*MEN1*) gene that encodes menin protein having three isoforms and is located on chromosome 11 (11q13.1) is classically known for germline inactivating mutations in several endocrine tumors [6–8]. The functional importance of menin has been thoroughly defined in previous studies, highlighting its decisive role in number of epigenetic regulations, DNA repair and metabolic pathways [9, 10]. *MEN1* has an enigmatic character and can function as an oncogene in cancers including leukaemia, prostate cancer, and hepatocellular carcinoma or as a tumor suppressor in endocrine tumors [11, 12]. Apart from mutational anomalies, expressional variations of *MEN1* are widely recognised in *MEN1* related disorders. Patients with overexpression of *MEN1* in prostate and hepatocellular carcinoma reportedly show poor survival when compared to patients with low *MEN1* expression [13, 14]. Also, the oncogenic behaviour of *MEN1*, through its direct interaction with MLL to promote leukemogenesis is well documented in prior studies [15]. *MEN1* deficiency is widely associated with an increased risk of developing breast cancer in female patients with *MEN1* syndrome [16]. However, a paradoxical role of menin is reported in sporadic breast cancer cases, where it shows proliferative function and is also linked with resistance to drug and endocrine therapy [16–18].

Although research in this field is ongoing, the role of *MEN1* in the onset and progression of breast cancer is not fully known. The expression of *MEN1* in the mammary glands may be controlled by the level of released hormones, such as prolactin, and have a negative response in the process of milk protein synthesis via PI3K/Akt/mTOR [19]. Disruption in hormonal balance due to *MEN1* mutations or its altered expression can potentially contribute to breast cancer development. In recent studies, direct interaction of menin with AF2 domain of ERα in estrogen positive cell line has been reported and it might promote ERα dependent transcription and proliferation. Moreover, for antiestrogen therapy drugs that bind to the AF2 domain of the ERα, menin’s interaction with this region could be the reason in patients with *MEN1* overexpression to develop resistance to adjuvant therapy [17, 20]. Apart from being co-activator for ERα, it also regulates expression of ESR in ER+ breast cancer cell that further supports its proliferative function in sporadic breast cancer cases [21].

The previously published literature signifies the distinctive functions of *MEN1* in breast cancer patients and also provides scope to evaluate its anomalous expression and connection with clinical parameters. Thereby our study targets to untangle *MEN1* expression at mRNA and protein level and also evaluate its epigenetic and polymorphic alterations in sporadic breast cancer patients. The findings of our study provide better insight in understanding the clinical significance of *MEN1* gene in the breast cancer cases.

## Material and methods

### Study samples

This study includes 142 clinically confirmed sporadic breast cancer cases that are genetically unrelated Indian females of age 20 to 79 years and with breast tumor as the primary site. The patients who have received chemotherapy or radiotherapy or had multiple tumors were excluded from the study. At the time of surgery aliquot of tumor along with adjacent normal tissues were taken in PBS, RNA later and formalin for further processing. The written informed consent was taken from the patients volunteering to participate in the study and the clinicopathological parameters considered at time of diagnosis were procured from patient’s record maintaining the patient’s confidentiality. The ethical approval to conduct this research was provided by Institute Ethics Committee, AIIMS, New Delhi (IEC-849/03.12.2021) and Institutional Ethics Committee, JMI, New Delhi (25/7/236/JMI/IEC/2019). All the experiments were conducted in the year 2022 to 2023 after obtaining the ethical approval from both the institutions and strictly following the ethical guidelines. The enrolment details and clinical profile of the patients are provided in the S1 Table.

### mRNA expression analysis

RNA was isolated from the tissue samples stored in RNA later at -80°C. Tissue were homogenized in TRIZOL using the homogenizer followed by the phase separation, precipitation and washing step as instructed in the manufacturer’s protocol. The obtained RNA pellet was dissolved in the RNAase free water and was treated with DNAse to eliminate any DNA contamination. The purity and the concentration of isolated RNA was assessed through nanodrop spectrophotometer at 260/280 ratio. RNA samples having purity ~2.0 were used for cDNA synthesis. cDNA were constructed using Verso cDNA synthesis kit, 1000 ng of RNA was used per 20 ul reaction.

The constructed cDNA was subjected to Real Time Polymerase Chain Reaction (RT-PCR) using specific primers designed for MEN1 and ACTB was taken as internal control (Table 1). The RT-PCR reaction was carried out using KAPA SYBR® FAST (cat no: KK4610) master mix, following the steps provided in manufacturer’s protocol. The RT-PCR data was analyzed by the comparative Ct method using formula: Fold Change = 2^-ΔΔCt^; ΔΔCt = (^Ct^targeted gene−^Ct^ACTB) targeted sample—(^Ct^targeted gene−^Ct^ACTB) calibration sample.

**Table 1:** List of primer sequences used in the study.

Primer	Primer sequence	PCR Product size (bp)	Annealing temperature (°C)
Methylation Primers			
*MEN1* Methylation	F 5′-TTTTTTAGAAGGTATTGCGGGTAC-3′	161	53
	R 5′-CCGCTAAACCTAAAAATAATAACGA-3′		
*MEN1* Unmethylation	F 5′-TTTTTAGAAGGTATTGTGGGTATGT-3′	160	54
	R 5′-CCACTAAACCTAAAAATAATAACAAA-3′		
Mutation Primers			
*MEN1* exon 8	F 5′-TTGCTTTCTTCCTCTGGGCTG-3′	396	57
	R 5′-CCTGCCATCCCTAATCCCGT-3′		
*MEN1* exon 9	F 5′-TGAGTAAGAGACTGATCTGTGCC-3′	276	60
	R 5- AAGTCTGACAAGCCCGTGGC-3′		
*MEN1* exon 10	F 5′-CAACCTTGCTCTCACCTTGCTC-3′	703	60
	R 5′- GCCCTGGGTTCTGAGCTGGAGAA-3′		
Expression Primers			
*MEN1*	F 5′-GCTGGCTGTACCTGAAAGGA-3′	257	58
	R 5′-CTTGTGGTAGAGGGTGAGTG-3′		
*ACTB*	F 5′-GTCATTCCAAATATGAGATGCGT-3′	121	58
	R 5′-GCTATCACCTCCCCTGTGTG-3′		

### DNA isolation

Genomic DNA was isolated by homogenizing 50–100 ug of tissue in the tissue lysis buffer and overnight incubation at 45°C [22]. Further, Phenol-chloroform method was used for DNA extraction from the tissue lysate. The qualitative and quantitative analysis of the obtained genomic DNA was done through gel electrophoresis using 0.7% agarose gel. The purity of the DNA was confirmed by nanodrop spectrophotometer by taking absorbance ratio at 260/280 A. The isolated genomic DNA having purity ~1.8 were used for further experiments.

### MS-PCR

**Fig 1:**
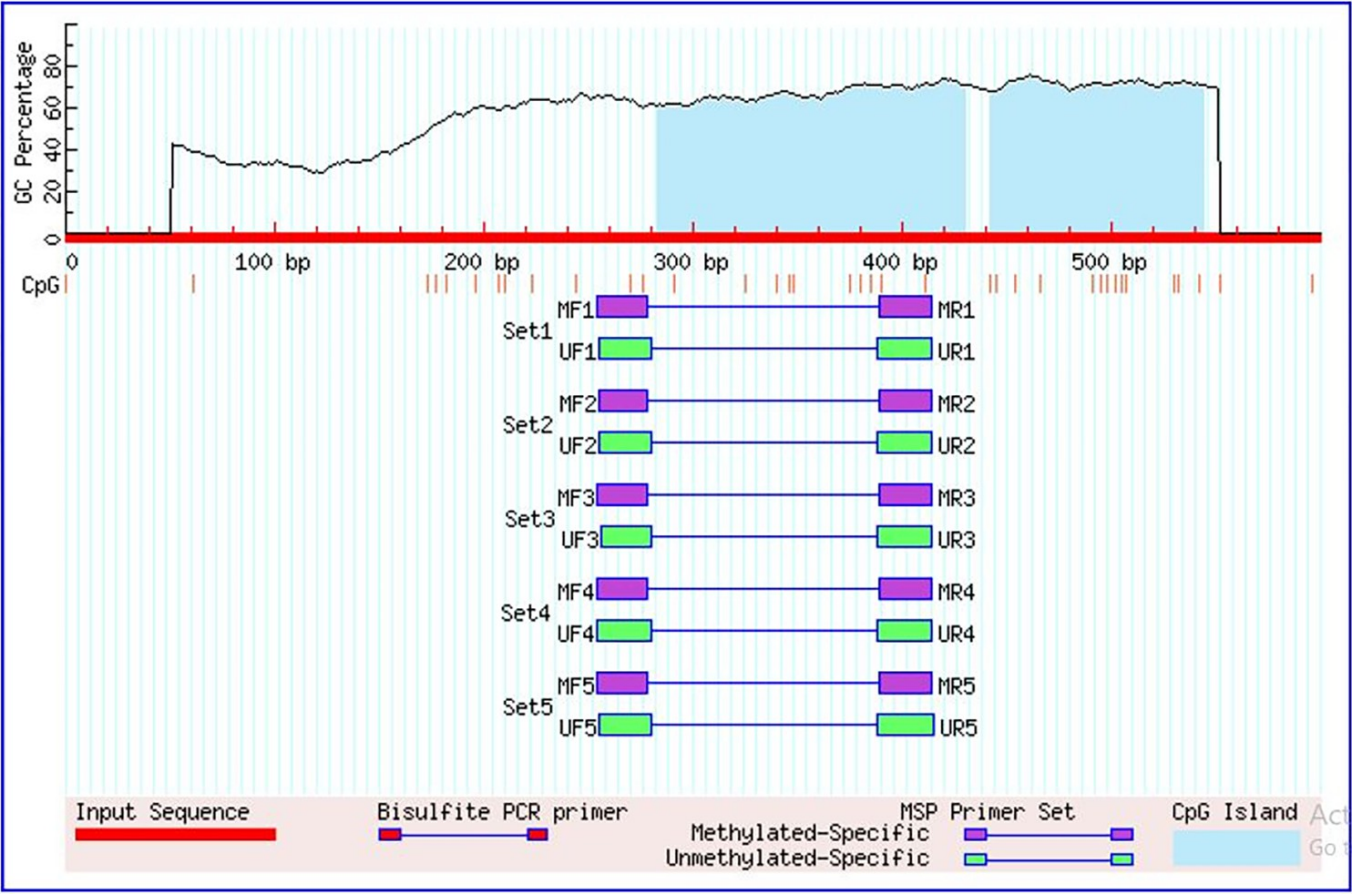
Graphical representation of CpG islands in the *MEN1* promoter region taken from MethPrimer.

To determine the promoter methylation status of *MEN1* gene through Methylation specific PCR (MS-PCR), firstly genomic DNA was bisulfite modified using EZ DNA Methylation-Lightning Kit (cat: D5030). Two sets of primers specific to methylated and unmethylated promoter region of *MEN1* gene were designed using methprimers (Table 1) (Fig 1). The PCR reaction of 25 ul was prepared containing 100 ng of bisulfite-converted DNA, 1.5 mM MgCl2, 200 μM of each deoxynucleotide triphosphates (dNTPs: dATP, dCTP, dGTP, and dTTP), 0.5 μM of each forward and reverse oligonucleotide primers, 1 x PCR buffer, and 1 unit of Hot Start Taq DNA Polymerase (Qiagen, Hilden, Germany). The PCR amplification of bisulfite modified DNA was carried out by using following the standardized protocol of our laboratory [23, 24]. The qualitative analysis of PCR product was done by gel electrophoresis using 2% agarose gel followed by visualization under UV-transilluminator.

### Immunohistochemistry

Differential expression of menin was analyzed through immunohistochemistry using specific antibody for menin (A500-003A). Formalin fixed tissue blocks of breast cancer tissue were prepared and sectioned on poly-L-lysine coated slides. Different grades of xylene were used for deparaffinizing the sections and then rehydrated through series of ethanol. 0.3% hydrogen peroxide was used to block endogenous peroxidase activity, further Ag retrieval was done through boiling in citrate buffer. The slides were subjected to overnight incubation at 4°C with primary antibody at dilution 1:200 followed by incubating with secondary antibody at 37°C for 1 hour. 3,3’- Diaminobenzidine (DAB) treatment was given for visualizing the antibody binding site and Hematoxylin was used for counter staining.Normal breast tissue were considered as the positive control and the sections processed following identical steps excluding primary antibody incubation served as negative control. The immunohistological staining was then interpreted by expert histopathologists and were graded as follows: (a) no expression- 0%, (b) mild expression- 1% - 10%, (c) moderate expression- 10% - 50%, (d) high expression- >50% [25].

### Western blotting

Total protein was isolated from tissue samples using RIPA buffer. The quantification of the protein was done through Bradford method using BSA concentration as the standard curve. Equal amount protein (30μg) was resolved on 10% SDS gel and was transferred to nitrocellulose membrane. One hour blocking in fat free milk was done followed by overnight primary antibody incubation for menin (D45B1) and control gene beta actin (sc-4778). After 3 times washing in TBST, membrane was incubated for 1 hour in suitable HRP conjugated secondary antibodies. Clarity western ECL substrate (cat: 170–5060) was used for protein detection by chemi-luminescence (Bio Rad).

### Automated DNA sequencing

*MEN1* mutations are prominently known in human cancers. *MEN1* gene comprises of total 10 exons of which exon 8 (136bp), exon 9 (165bp) and exon 10 (1,301bp) of *MEN1* gene are identified for their critical relevance in menin interaction with various proteins and also coding nucleus localizing signals. The mentioned exons were amplified using specific primers and PCR product were purified using Qiagen MinElute PCR Purification kit (Cat. No.28004). The amplified products were subjected to direct sequencing at Macrogen Inc., South Korea Lab using both forward and reverse primers. The sequencing results were analyzed using the software clustal omega. The sequencing was repeated to rule out any contamination or false results.

### METABRIC data analysis

METABRIC data set comprises of the clinical profiles, survival data, copy number variants, expression and SNP genotypes of the patients participating in the METABRIC trial. We analyzed the *MEN1* expression in the breast cancer patients included in the METABRIC trial data set available at cBioPortal and compared the survival data of the participants with low *MEN1* and high *MEN1* expression. Survival curve was plotted using graph pad prism.

### Statistical analysis

The statistical correlation of our molecular findings and clinical parameters was done using SPSS-IBM (version 22.0) and graph pad prism. The mRNA expression data in the study have been expressed as mean ± standard error of mean. Non-parametric test i.e. Wilcoxon signed-ranked test and Kruskal wallis test were performed to evaluate the significant difference between mRNA expression of *MEN1/ACTB* and compare the mRNA expression with clinical parameters respectively. The correlation of protein expression and methylation status with clinical parameters was performed using chi square test and the *p* value <0.05 is considered to be significant.

## Results

### Clinical profiling of the enrolled patients

In current study, breast tissue samples from female breast cancer patients (N = 142) were taken at the time of surgery. Majority of the patients enrolled in the study are postmenopausal (87/142), indicating a higher frequency of breast cancer among postmenopausal females. The status of the hormone receptors ER, PR, and Her2 neu of the recruited patients was screened, and were categorized into molecular subtypes accordingly. Most of the patients (101/142) were at the advanced stage (III and IV), signifying poor diagnosis of the disease (Table 2).

**Table 2:** Clinical profiling of the patients included in the study.

Characteristics	Cases (%)
**Tissue**	
Normal	142 (100)
Tumor	142 (100)
**Age**	
≤50	80 (56.34)
>50	62 (43.66)
**Menopausal status**	
Premenopausal	55 (38.7)
Postmenopausal	87 (61.3)
**Age at menopause**	
≤45	45 (51.72)
>45	42 (48.28)
**Estrogen receptor status**	
Negative	81 (57)
Positive	61(43)
**Progesterone receptor status**	
Negative	91 (64.1)
Positive	51 (35.9)
**Her2 neu status**	
Negative	89 (62.7)
Positive	53 (37.3)
**Lymph node status**	
Negative	39 (27.5)
Positive	103 (72.5)
**Tumor size**	
≤5	46 (32.4)
>5	96 (67.6)
**Histological grade**	
(I + II)	85 (59.9)
(III + IV)	57 (40.1)
**Clinical stage**	
(I+II)	41 (28.9)
(III + IV)	101 (71.1)
**Molecular subtype**	
Luminal A	39 (27.5)
Luminal B	25 (17.6)
Her2 neu enriched	28 (19.7)
TNBC	50 (35.2)

### Upregulated expression of *MEN1* mRNA in breast tumors

**Fig 2:**
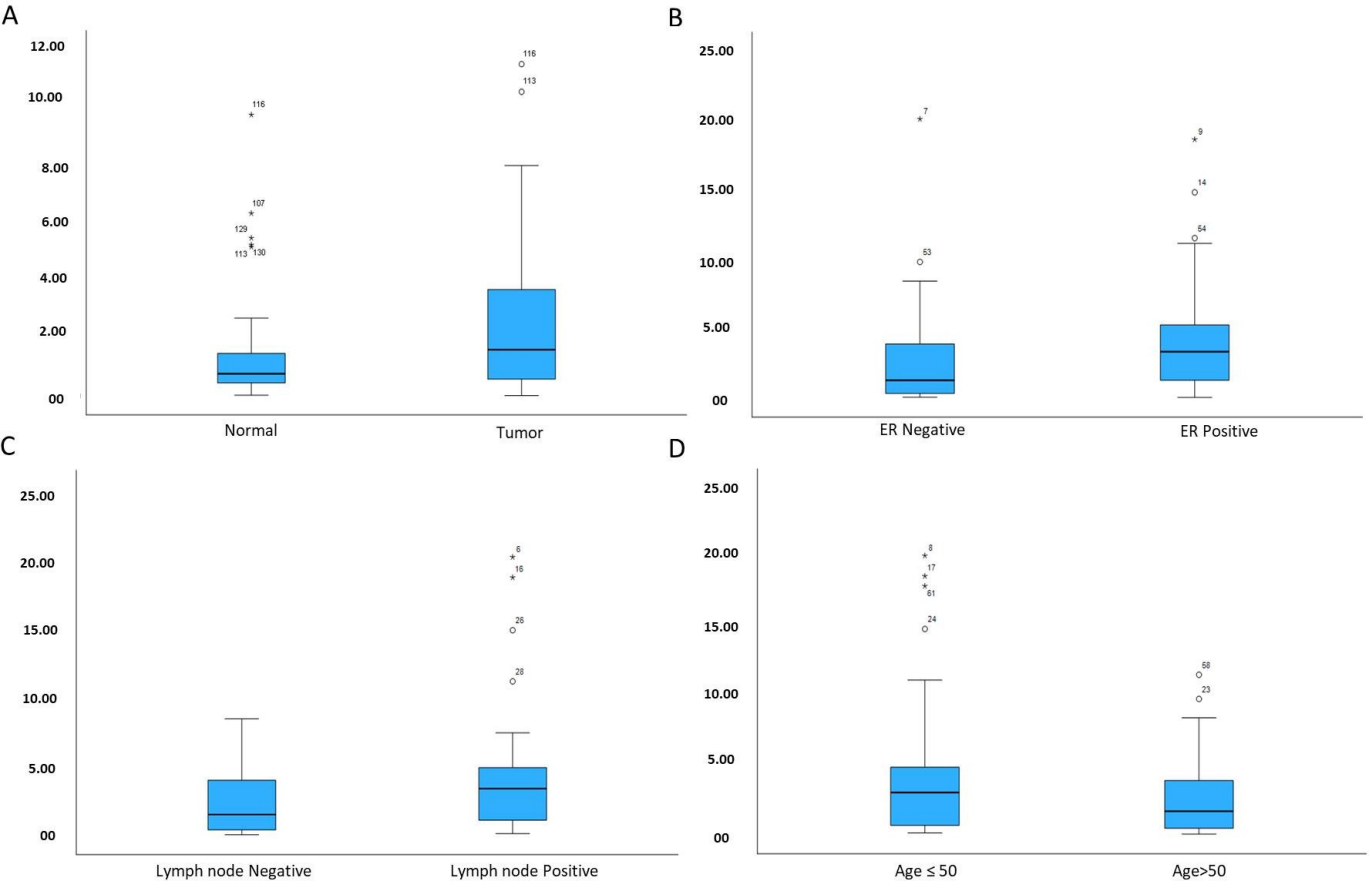
Box plots representing significant change in expression of *MEN1* mRNA (A) Breast tumor and adjacent normal tissue. (B) ER negative and ER positive cases (C) Lymph node negative and lumph node positive cases (D) Age upto 50 and age more than 50.

Our analysis of mRNA expression using real-time PCR shows that *MEN1* mRNA is upregulated in breast tumor samples as compared to the nearby normal tissue samples. The housekeeping gene *ACTB* was used to normalize the expression of *MEN1*, and a mean fold change of 5.17 was observed in the upregulated cases. Overall, 63.38% (90/142) cases exhibited the overexpression expression of *MEN1* at mRNA level and on correlating the elevated expression with clinical parameters; the significant association was observed with estrogen receptor status (*p* = 0.015), Age of the patient (*p* = 0.028) and lymph node status (*p* = 0.024). However, in our results we found that correlation of *MEN1* upregulation with molecular subtype (*p* = 0.118) and clinical stage (*p* = 0.341) of breast cancer is not significant (Table 3) (Fig 2).

**Table 3:** *MEN1* mRNA expression and its correlation with clinicopathological parameters of the patients.

Characteristics	Cases (%)	MEN1 mRNA expression relative to ACTB ± SE	*p* value
Normal	142 (100)	1.151 ± 0.1039	<0.001*
Tumor	142 (100)	2.412 ± 0.1956	
**Age**			
≤50	80 (56.3)	4.070 ± 0.5165	0.028*
>50	62 (47.3)	2.636 ± 0.3442	
**Menopausal status**			
Premenopausal	55 (38.7)	3.591 ± 0.5323	0.870
Postmenopausal	87 (61.3)	3.348 ± 0.4267	
**Age at menopause**			
≤45	45 (51.72)	4.380 ± 4.9004	0.098
>45	42 (48.28)	2.665 ± 3.5694	
**Estrogen receptor status**			
Negative	81 (57)	2.890 ± 0.4465	0.015*
Positive	61(43)	4.180 ± 0.4835	
**Progesterone receptor status**			
Negative	91 (64.1)	3.142 ± 0.4085	0.149
Positive	51 (35.9)	3.983 ± 0.5656	
**Her2 neu status**			
Negative	89 (62.7)	3.353 ± 0.4136	0.444
Positive	53 (37.3)	3.596 ± 0.5598	
**Lymph node status**			
Negative	39 (27.5)	2.531 ± 0.4073	0.024*
Positive	103 (72.5)	3.790 ± 0.4269	
**Tumor size**			
≤5	46 (32.4)	3.326 ± 0.5190	0.866
>5	96 (67.6)	3.500 ± 0.4250	
**Histological grade**			
(I + II)	85 (59.9)	3.469 ± 0.3970	0.790
(III + IV)	57 (40.1)	3.407 ± 0.5818	
**Clinical stage**			
(I+II)	41 (28.9)	2.763 ± 0.4176	0.341
(III + IV)	101 (71.1)	3.720 ± 0.4329	
**Molecular subtype**			
Luminal A	39 (27.5)	3.657 ± 0.5824	0.118
Luminal B	25 (17.6)	4.399 ± 0.8044	
Her2 neu enriched	28 (19.7)	3.064 ± 0.7640	
TNBC	50 (35.2)	3.014 ± 0.5840	

### Loss of promoter methylation in cases with *MEN1* overexpression

**Fig 3:**
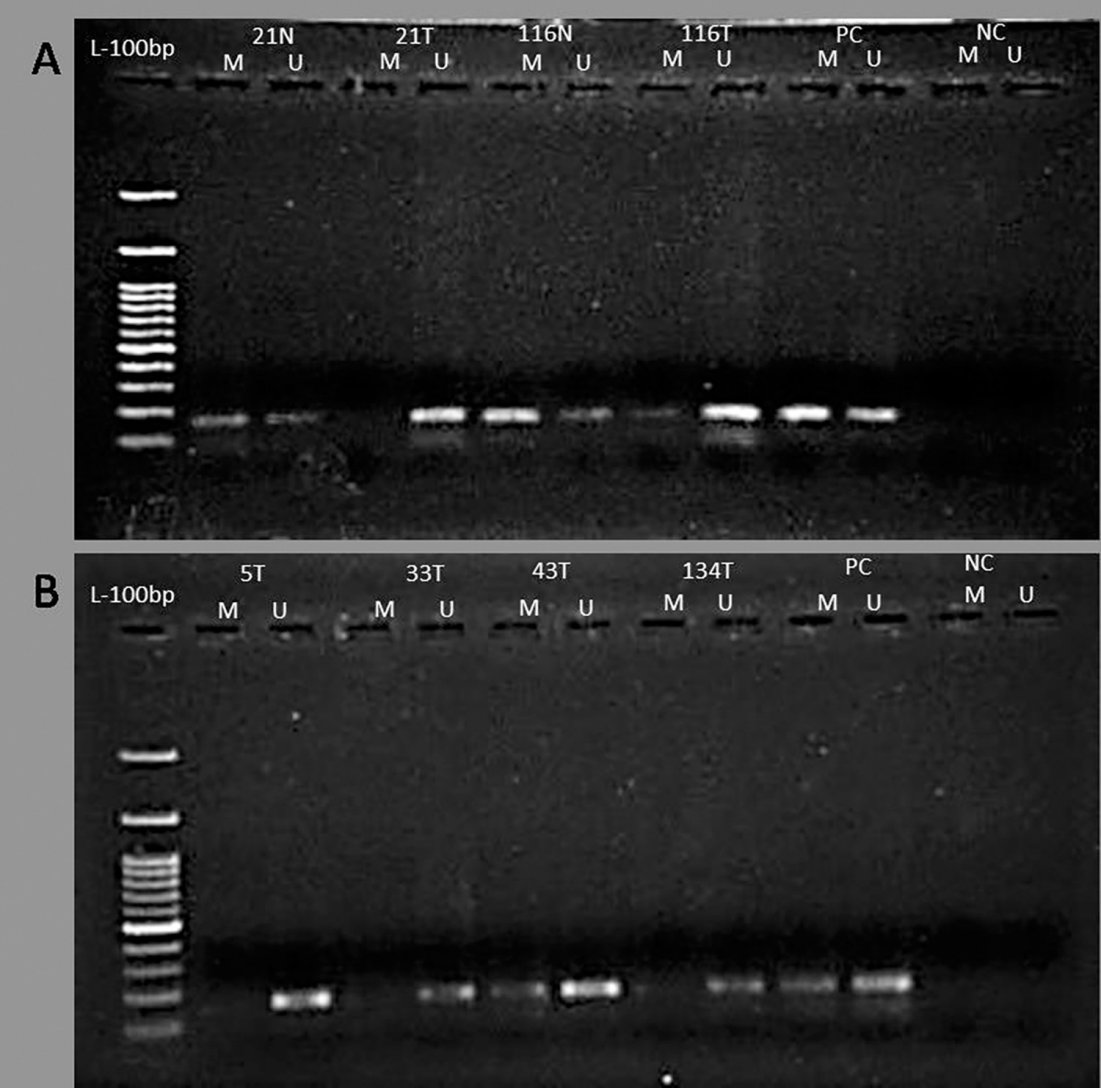
(A) Representative Agarose gel picture for MS-PCR showing promoter methylation (product size: M = 161) and unmethylation status (product size: UM = 160) of *MEN1* in tumor tissue and adjacent normal breast tissue. (B) MS-PCR results exhibiting the *MEN1* promoter methylation and unmethylation status of breast tumor tissue.

MS-PCR of bisulfite modified DNA revealed unmethylated CpGs in the *MEN1* promoter region of 53.52% (76/142) breast tumor samples, with the majority (68.42%) having elevated menin expression. The lack of promoter methylation was found to have a significant correlation (*p*<0.001) with *MEN1* expression, with 72.37% (55/76) cases exhibiting upregulated *MEN1* expression. Furthermore, our promoter methylation study shows aberrant promoter methylation in 29 cases, out of which only 09 cases had *MEN1* mRNA overexpression. In addition, 37 cases had no change in promoter methylation status and were considered as unaltered. In advanced stages III and IV of breast cancer, a strong correlation (*p* = 0.034) with unmethylated promoter region was observed, with 80.26% (61/76) cases having unmethylated *MEN1* promoter region. Also, the age of the patients at menopause exhibited significant association (*p* = 0.009) and 34.48% (30/45) early menopause cases had unmethylated CpG’s in *MEN1* promoter region (Table 4) (Fig 3).

**Table 4:** Correlation of *MEN1* promoter methylation status with clinicopathological parameters of the patients.

Characteristics	Cases (%)	Unmethylated (%)	Methylated (%)	Unaltered (%)	*p* value
**Age**					
≤50	80 (56.3)	43 (30.28)	17 (11.97)	20 (14.08)	0.932
>50	62 (47.3)	33 (23.24)	12 (08.45)	17 (11.97)	
**Menopausal status**					
Premenopausal	55 (38.7)	24 (16.90)	15 (10.56)	16 (11.27)	0.134
Postmenopausal	87 (61.3)	52 (36.62)	14 (9.86)	21 (14.79)	
**Age at menopause**					
≤45	45 (51.72)	30 (34.48)	10 (11.49)	5 (05.75)	0.009*
>45	42 (48.28)	22 (25.29)	4 (04.60)	16 (18.39)	
**Estrogen receptor status**					
Negative	81 (57)	41 (28.87)	19 (13.38)	21 (14.79)	0.563
Positive	61(43)	35 (24.65)	10 (07.04)	16 (11.27)	
**Progesterone receptor status**					
Negative	91 (64.1)	49 (34.51)	19 (13.38)	23 (16.20)	0.956
Positive	51 (35.9)	27 (19.01)	10 (07.04)	14 (09.86)	
**Her2 neu status**					
Negative	89 (62.7)	44 (30.99)	21 (14.79)	24 (16.90)	0.369
Positive	53 (37.3)	32 (22.54)	08 (05.63)	13 (09.15)	
**Lymph node status**					
Negative	39 (27.5)	17 (11.97)	09 (06.34)	13 (09.15)	0.322
Positive	103 (72.5)	59 (41.55)	20 (14.08)	24 (16.90)	
**Tumor size**					
≤5	46 (32.4)	20 (14.08)	12 (08.45)	14(09.86)	0.240
>5	96 (67.6)	56 (39.44)	17 (11.97)	23 (16.20)	
**Histological grade**					
(I + II)	85 (59.9)	43 (30.28)	17 (11.97)	25 (17.61)	0.529
(III + IV)	57 (40.1)	33 (23.24)	12 (08.45)	12 (08.45)	
**Clinical stage**					
(I+II)	41 (28.9)	15 (10.56)	12 (08.45)	14 (09.86)	0.034*
(III + IV)	101 (71.1)	61 (42.96)	17 (11.97)	23 (16.20)	
**Molecular subtype**					
Luminal A	39 (27.5)	21 (14.78)	7 (04.93)	11 (07.75)	0.779
Luminal B	25 (17.6)	15 (10.56)	3 (02.11)	7 (04.93)	
Her2 neu enriched	28 (19.7)	16 (11.27)	5 (03.52)	7 (04.93)	
TNBC	50 (35.2)	24 (16.90)	14 (09.86)	12 (08.45)	

### MEN1 protein expression and its association with clinicopathological parameters in breast cancer

**Fig 4:**
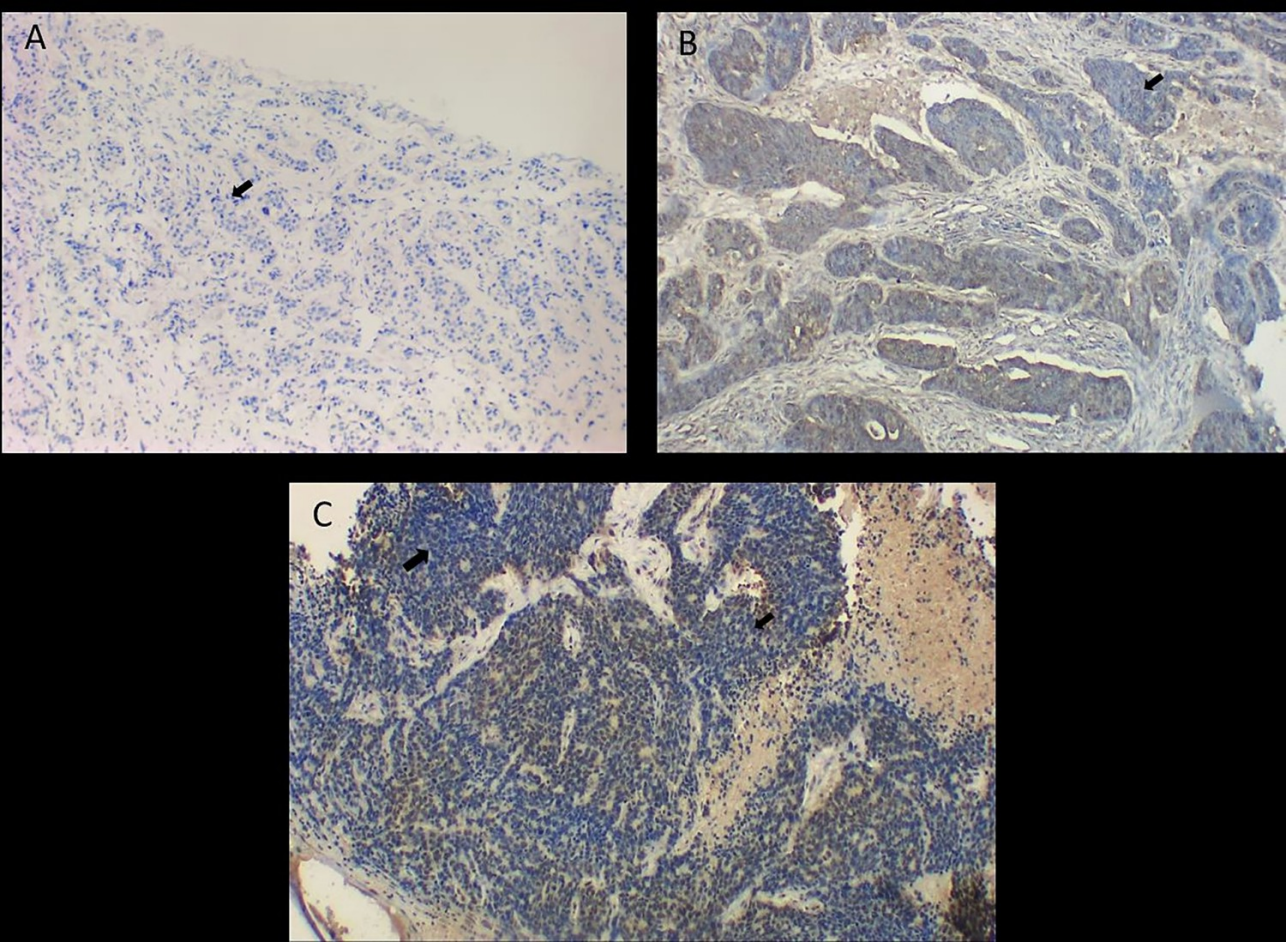
Representative panel of immunohistochemical images taken at 20X magnification for MEN1 protein detection in breast tissue (A) Normal Breast tissue (B) Breast cancer tissue showing low expression of menin protein (C) Breast cancer tissue with high expression of menin protein.

**Fig 5:**
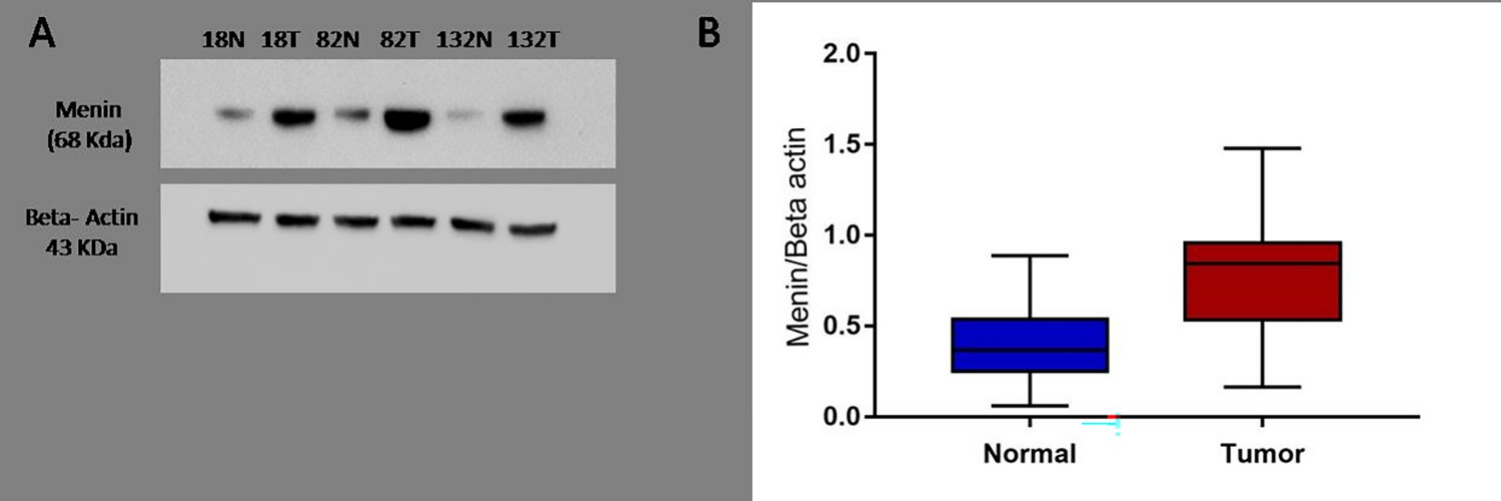
MEN1 protein expression analysis through western blot (A) elevated expression of menin in tumor tissue as compared to its paired normal breast tissue (B) comparative analysis of menin/Beta actin expression in normal and breast tumor tissues.

Immunohistochemistry and western blotting results were compared and analysed to determine the expression of the *MEN1* gene at the protein level. Menin was found to be overexpressed in 86 of 142 patients, with its predominant nuclear localization. The Menin protein was elevated in 60.56% of cases, with its significant upregulation in breast tumor as compared to normal tissue. Our findings indicate a strong association between increased Menin expression and ER positive cases (*p* = 0.002), with 46 of 61 ER positive cases showing elevated Menin expression (Table 5) (Figs 4 and 5).

**Table 5:** MEN1 protein expression and its correlation with clinicopathological parameters of the patients.

Characteristics	Cases (%)	Low Menin (%)	High Menin (%)	*p* value
**Age**				
≤50	80 (56.3)	31 (21.83)	49 (34.50)	0.849
>50	62 (47.3)	25 (17.60)	37 (26.05)	
**Menopausal status**				
Premenopausal	55 (38.7)	19 (13.38)	36 (25.35)	0.343
Postmenopausal	87 (61.3)	37 (26.05)	50 (35.21)	
**Age at menopause**				
≤45	45 (51.72)	16 (18.39)	29 (33.34)	0.173
>45	42 (48.28)	21 (24.14)	21(24.14)	
**Estrogen receptor status**				
Negative	81 (57)	41 (28.87)	40 (28.16)	0.002*
Positive	61(43)	15 (10.56)	46 (32.39)	
**Progesterone receptor status**				
Negative	91 (64.1)	39 (27.46)	52 (36.61)	0.265
Positive	51 (35.9)	17 (11.97)	34 (23.94)	
**Her2 neu status**				
Negative	89 (62.7)	38 (26.76)	51 (35.91)	0.303
Positive	53 (37.3)	18 (12.67)	35 (24.64)	
**Lymph node status**				
Negative	39 (27.5)	19 (13.38)	20 (14.08)	0.164
Positive	103 (72.5)	37 (26.05)	66 (46.47)	
**Tumor size**				
≤5	46 (32.4)	20 (14.08)	26 (18.30)	0.495
>5	96 (67.6)	36 (25.35)	60 (42.25)	
**Histological grade**				
(I + II)	85 (59.9)	33 (23.23)	52 (36.62)	0.855
(III + IV)	57 (40.1)	23 (16.19)	34 (23.94)	
**Clinical stage**				
(I+II)	41 (28.9)	21 (14.78)	20 (14.08)	0.067
(III + IV)	101 (71.1)	35 (24.64)	66 (46.47)	
**Molecular subtype**				
Luminal A	39 (27.5)	14 (9.85)	25 (17.60)	0.085
Luminal B	25 (17.6)	5 (3.52)	20 (14.08)	
Her2 neu enriched	28 (19.7)	12 (8.45)	16 (11.26)	
TNBC	50 (35.2)	25 (17.60)	25 (17.60)	

### *MEN1* is not mutated in Indian breast cancer patients

**Fig 6:**
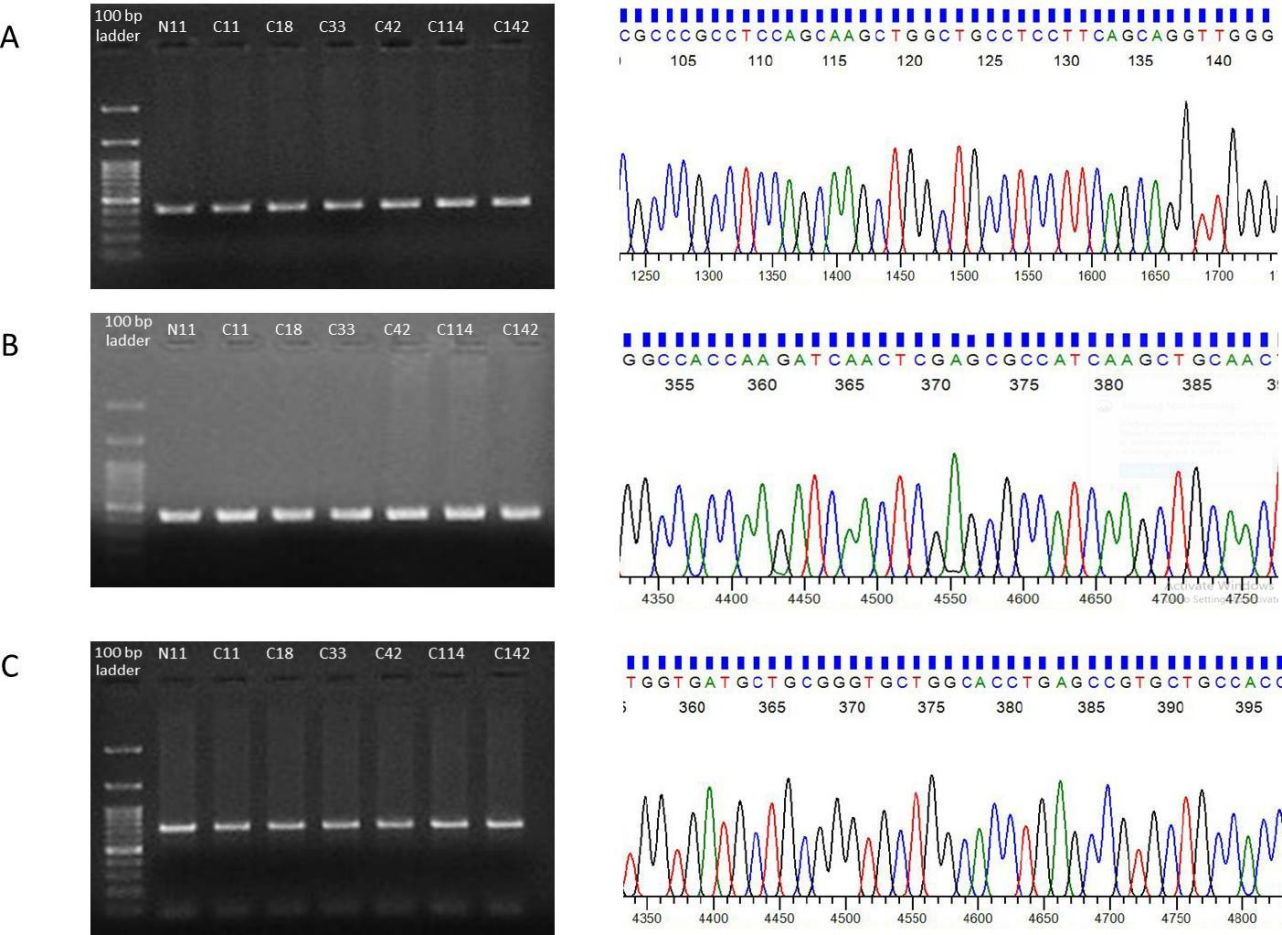
Representative agarose gel images showing PCR amplification and electropherogram of MEN1 exon 8, exon 9 and exon 10 for mutation analysis.

*MEN1* mutations are frequent and aggressive C-terminus of MEN1 protein forms the finger domain and contains three nucleus localizing sequences. The disruption in C-terminus is known to have a vital role in tumorigenesis [9, 26, 27]. However, our automated sequencing analysis did not show any mutation in the exons coding this domain of *MEN1* gene (Fig 6).

### High *MEN1* expression is associated with poor survival

**Fig 7:**
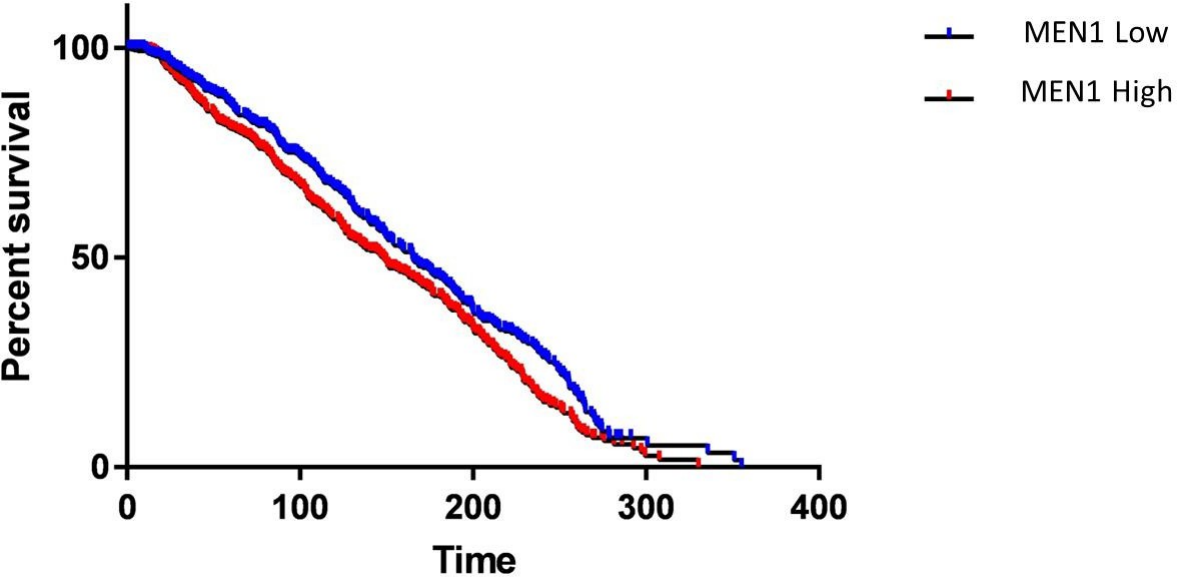
METABRIC data analysis to compare survivalship of the patients with low *MEN1* and high *MEN1* expression.

The METABRIC data analysis to compare the survival among the patients with low *MEN1* and high *MEN1* expression was done [28]. Poor survival was observed amongst the participants having higher expression of *MEN1* gene when compared to participants with low *MEN1* expression (Fig 7).

## Discussion

Even after rigorous research and advancement made in diagnosis and treatment, the steady rise in breast cancer incidence has compelled clinicians and researchers to revisit our traditional approaches in managing this disease [29]. Even though most of the patients share common histological features at the time of diagnosis, the underlying molecular aspects of the disease leads to varied clinical outcomes in response to traditional therapies [30, 31]. Moreover, these traditional therapies are much focused on ER, PR and Her2 expression status to treat the disease rather than evaluating complex expression patterns of related genes. To ensure more assertive clinical outcomes and survival of the patients, personalized approach of treatment with better understanding of molecular drivers of chief pathways involved in the progression of breast cancer is needed [32–34].

Earlier studies on women with sporadic breast cancer and those with the MEN1 syndrome have underlined the contradicting role of *MEN1* in the disease. The anti-proliferative function of *MEN1* is reported in normal mammary epithelium cells and females with MEN1 syndrome are at high risk of developing cancer. However, the contradictory role of *MEN1* in sporadic breast cancer cases is observed where it is linked to tumorigenesis through coactivation of ERα [35–37]. While the molecular functions of *MEN1* are being widely explored in previous researches, we aimed to evaluate the *MEN1* gene expression pattern and determine its relevance to clinical parameters in 142 sporadic breast cancer patients.

The findings of our study reveal the upregulated expression of *MEN1* mRNA in nearly 63% of breast cancer cases. Interestingly, 46 of 61 ER+ patients included in the study had elevated levels of *MEN1* mRNA, showing a strong correlation between *MEN1* mRNA overexpression and ER+ status. Further, when the protein expression was evaluated with IHC and western blotting, similar outcome was observed. Almost 60% cases exhibited the elevated expression of menin protein with its prominent nuclear localization and showed significant association with ER status of the patients. *MEN1* is majorly a nuclear protein and is known to be crucial regulator of gene transcription and cellular pathways [38]. Its direct interaction with various histone modifiers like *PRMT5*, *MLL*, and *SUV39H1* is well established in the previous studies. Also, *MEN*1 interacts with *NFKβ*, *JunD*, *cMyc* transcription factors and control the expression of associated genes [10, 39]. These diverse interactions of *MEN1* are responsible for its dual behavior in tumorigenesis and also its tissue specific functioning. In the recently published literature, probable role of *MEN1* in *ESR* expression is reported in ER+ breast cancer cell [18, 20, 21]. These connections indeed support our findings and *MEN1* could be a key player of cellular proliferation in ER+ cells.

The upregulated expression of *MEN1* mRNA was also found to be significantly associated with lymph node status and age of the patients. The positive lymph node score of axillary lymph nodes is considered as important prognostic marker in breast cancer and is also known to have critical role in tumor free survival of the patients [40]. The role of menin in epithelial to mesenchymal transition is reported previously and can lead to metastatic response via TGF-β/Menin/C/EBPβ regulatory axis [41]. In most of the patients included in our study, higher *MEN1* mRNA expression was observed in lymph node positive cases, indicating its possible role in poor survival of the patients. To further investigate epigenetic or polymorphic alteration that could possibly be involved in the anomalous expression of *MEN1* in breast tumors, MS-PCR and Sanger sequencing was performed. Mutations in the *MEN1* gene can cause menin function to be lost or altered, compromising the normal regulation of cell growth and division [9, 42]. The C terminal of MEN1 protein has nucleus localizing sequences that are critical for its nuclear import; however there are very limited instances where *MEN*1 mutations in cases of sporadic breast cancer are reported [9, 43]. We did not find any alteration in these sequences in breast cancer cases included in our study. To ensure continuous growth, cancer cells undergo multiple changes to alter gene expression programming of which DNA methylation alterations across the genome have major role. DNA methylation status is known to have strong connection with mRNA levels [44, 45]. Our promoter methylation study for *MEN1* gene depicts the hypomethylation in majority of the cases and when compared with the clinical parameters of the patients, majority of the unmethylated cases were of advanced clinical stages. In persistence, when the promoter hypomethylation was compared with *MEN1* mRNA and protein expression, a significant correlation was seen.

The critical association of *MEN1* with Androgen receptor signalling pathway and its oncogenic role in prostate cancer is well documented. Its higher expression is reported to be linked with poor survival and developing resistance to the treatment in various malignancies including hepatocellular carcinoma and prostate cancer [11–13, 46]. We further evaluated the significance of *MEN1* expression in survival of the breast cancer patients. METABRIC data was analyzed and the patients with higher expression of *MEN1* were found to have poor survival in comparison to cases with low *MEN1* expression. In future, case studies could be designed on larger population to evaluate the differential expression of *MEN1* gene in different phenomenon like disease free survival, metastasis and resistance to hormone and drug therapy. This will give better insight in understanding clinical significance of *MEN1*in breast cancer patients.

## Conclusion

In conclusion, our study demonstrates the higher expression of *MEN1* in sporadic breast cancer patients. Previous researches indicated the enigmatic role of *MEN1* in breast cancer, where its positive association is evident with *ERα* and *ESR* [21]. The overexpression of the *MEN1* gene in Indian breast cancer patients is reported for the first time in our study and it exhibits substantial correlation with the ER+ status of the patients. In most of the cases *MEN1* promoter region was unmethylated and had significant association with high expression of *MEN1* gene. We also report that *MEN1* mutations, especially at NLS are not prominent in Indian breast cancer patients included in our study. Our findings can be pioneer for further research to determine if *MEN1* plays a tumor suppressive or oncogenic role in breast cancer.

## Supporting information

S1 Table Clinical profile of patients included in the study (2015–2022). (PDF)

S1 File (DOCX)

S2 File (ZIP)
