# Supplementary material for: Evaluating the role of MEN1 gene expression and its clinical significance in breast cancer patients
Source: PLoS One. 2023 Jul 12;18(7):e0288482. doi: 10.1371/journal.pone.0288482 (PMC10337982; doi:10.1371/journal.pone.0288482)
Supplement: S1 Table — (PDF) [file pone.0288482.s001.pdf]

# Clinical profile of patients included in the study (2015-2022)

| S.No. | IRCH | Age | Menopause Age | Menopause status | ER status | PR status | Her2 Neu | Mol subtype | tumor size | lymph node status | Histological grade | Clinical stage |
|---|---|---|---|---|---|---|---|---|---|---|---|---|
| 1 | 163898 | 52 | 46 | Post menopausal | Negative | Negative | Negative | TNBC | >5 | Negative | 2 | 3b |
| 2 | 168619 | 30 | Pre | Pre menopausal | Negative | Negative | Negative | TNBC | >5 | Positive | 3 | 3b |
| 3 | 164852 | 36 | Pre | Pre menopausal | Positive | Positive | Positive | Luminal B | >5 | Positive | 3 | 4 |
| 4 | 163828 | 60 | 50 | Post menopausal | Positive | Positive | Negative | Luminal A | <5 | Negative | 2 | 2a |
| 5 | 161323 | 51 | 48 | Post menopausal | Positive | Negative | Negative | Luminal A | >5 | Positive | 3 | 3a |
| 6 | 168623 | 50 | 40 | Post menopausal | Positive | Negative | Negative | Luminal A | >5 | Negative | 1 | 3b |
| 7 | 164106 | 36 | Pre | Pre menopausal | Negative | Negative | Negative | TNBC | <5 | Negative | 3 | 4 |
| 8 | 166187 | 47 | Pre | Pre menopausal | Negative | Negative | Negative | TNBC | >5 | Positive | 2 | 3b |
| 9 | 163837 | 50 | 47 | Post menopausal | Negative | Positive | Positive | Luminal B | >5 | Positive | 3 | 3b |
| 10 | 168393 | 40 | 40 | Post menopausal | Negative | Positive | Negative | Luminal A | >5 | Negative | 2 | 2b |
| 11 | 165234 | 50 | 48 | Post menopausal | Negative | Negative | Positive | Her2+ | >5 | Positive | 3 | 3b |
| 12 | 172189 | 48 | Pre | Pre menopausal | Positive | Positive | Positive | Luminal B | <5 | Positive | 2 | 3a |
| 13 | 172521 | 39 | 38 | Post menopausal | Positive | Positive | Negative | Luminal A | >5 | Positive | 2 | 3a |
| 14 | 168462 | 52 | 41 | Post menopausal | Positive | Positive | Negative | Luminal A | >5 | Negative | 2 | 3b |
| 15 | 171806 | 50 | 42 | Post menopausal | Negative | Negative | Negative | TNBC | <5 | Positive | 2 | 2b |
| 16 | 176151 | 61 | 49 | Post menopausal | Negative | Positive | Positive | Luminal B | <5 | Negative | 2 | 2a |
| 17 | 175858 | 36 | Pre | Pre menopausal | Negative | Negative | Negative | TNBC | >5 | Positive | 2 | 3c |
| 18 | 167804 | 53 | 42 | Post menopausal | Negative | Negative | Positive | Her2+ | >5 | Positive | 2 | 4 |
| 19 | 176360 | 58 | 52 | Post menopausal | Negative | Negative | Positive | Her2+ | >5 | Positive | 3 | 3b |
| 20 | 170726 | 45 | 42 | Post menopausal | Positive | Positive | Positive | Luminal B | >5 | Positive | 1 | 3c |
| 21 | 175591 | 35 | 35 | Post menopausal | Negative | Negative | Negative | TNBC | >5 | Positive | 3 | 3b |
| 22 | 176582 | 53 | 51 | Post menopausal | Negative | Negative | Negative | TNBC | <5 | Negative | 2 | 2a |
| 23 | 170070 | 42 | Pre | Pre menopausal | Negative | Negative | Negative | TNBC | >5 | Positive | 3 | 3b |
| 24 | 172023 | 40 | Pre | Pre menopausal | Negative | Positive | Positive | Luminal B | >5 | Negative | 2 | 3b |
| 25 | 172977 | 44 | 42 | Post menopausal | Positive | Positive | Positive | Luminal B | >5 | Positive | 2 | 3b |
| 26 | 178777 | 71 | 43 | Post menopausal | Negative | Negative | Positive | Her2+ | <5 | Positive | 1 | 2b |

| | | | | | | | | | | | | | | |
|---|---|---|---|---|---|---|---|---|---|---|---|---|---|---|
| 27 | 177943 | 53 | | 46 | Post menopausal | Negative | Negative | Negative | TNBC | <5 | Positive | 3 | 2b | |
| 28 | 178314 | 60 | | 50 | Post menopausal | Negative | Negative | Positive | Her2+ | >5 | Positive | 2 | 3a | |
| 29 | 171969 | 48 | | 45 | Post menopausal | Positive | Positive | Negative | Luminal A | >5 | Negative | 2 | 3b | |
| 30 | 180724 | 45 | Pre | | Pre menopausal | Positive | Positive | Positive | Luminal B | >5 | Positive | 2 | 3a | |
| 31 | 178579 | 41 | Pre | | Pre menopausal | Negative | Negative | Positive | Her2+ | >5 | Negative | 2 | 2b | |
| 32 | 181909 | 50 | | 48 | Post menopausal | Positive | Positive | Positive | Luminal B | <5 | Positive | 2 | 3a | |
| 33 | 179673 | 45 | Pre | | Pre menopausal | Positive | Positive | Negative | Luminal A | >5 | Positive | 2 | 3c | |
| 34 | 180282 | 50 | Pre | | Pre menopausal | Negative | Negative | Negative | TNBC | >5 | Positive | 3 | 3a | |
| 35 | 183216 | 70 | | 45 | Post menopausal | Negative | Negative | Negative | TNBC | <5 | Positive | 2 | 2a | |
| 36 | 178316 | 52 | | 46 | Post menopausal | Negative | Negative | Positive | Her2+ | >5 | Positive | 3 | 3a | |
| 37 | 183441 | 44 | Pre | | Pre menopausal | Positive | Positive | Negative | Luminal A | <5 | Positive | 2 | 3c | |
| 38 | 174604 | 45 | Pre | | Pre menopausal | Positive | Positive | Positive | Luminal B | >5 | Positive | 1 | 3b | |
| 39 | 185306 | 44 | Pre | | Pre menopausal | Positive | Positive | Negative | Luminal A | <5 | Positive | 2 | 2b | |
| 40 | 177728 | 45 | | 41 | Post menopausal | Negative | Negative | Negative | TNBC | >5 | Negative | 2 | 3b | |
| 41 | 181686 | 53 | | 40 | Post menopausal | Positive | Negative | Positive | Luminal B | >5 | Positive | 2 | 3a | |
| 42 | 180073 | 37 | Pre | | Pre menopausal | Positive | Positive | Positive | Luminal B | <5 | Positive | 3 | 2b | |
| 43 | 184985 | 66 | | 48 | Post menopausal | Positive | Positive | Positive | Luminal B | <5 | Positive | 2 | 2b | |
| 44 | 184983 | 48 | | 48 | Post menopausal | Positive | Positive | Negative | Luminal A | <5 | Positive | 2 | 2b | |
| 45 | 186043 | 41 | Pre | | Pre menopausal | Negative | Negative | Positive | Her2+ | >5 | Negative | 2 | | 4 |
| 46 | 185925 | 60 | | 52 | Post menopausal | Positive | Negative | Positive | Luminal B | >5 | Positive | 2 | 3b | |
| 47 | 187461 | 50 | | 52 | Post menopausal | Positive | Negative | Negative | Luminal A | <5 | Negative | 2 | 2b | |
| 48 | 187449 | 69 | | 48 | Post menopausal | Negative | Negative | Positive | Her2+ | <5 | Positive | 2 | 3a | |
| 49 | 178838 | 45 | Pre | | Pre menopausal | Negative | Negative | Negative | TNBC | >5 | Positive | 1 | 3b | |
| 50 | 183714 | 56 | | 52 | Post menopausal | Negative | Negative | Positive | Her2+ | >5 | Positive | 2 | 3b | |
| 51 | 187622 | 59 | | 49 | Post menopausal | Negative | Negative | Positive | Her2+ | >5 | Positive | 2 | 3b | |
| 52 | 188006 | 44 | Pre | | Pre menopausal | Positive | Positive | Positive | Luminal B | <5 | Positive | 3 | 2a | |
| 53 | 189794 | 47 | Pre | | Pre menopausal | Positive | Positive | Negative | Luminal A | >5 | Negative | 2 | 2b | |
| 54 | 189499 | 60 | | 40 | Post menopausal | Negative | Negative | Positive | Her2+ | <5 | Positive | 2 | 2b | |
| 55 | 190564 | 34 | Pre | | Pre menopausal | Negative | Positive | Negative | Luminal A | >5 | Negative | 3 | 2b | |
| 56 | 191965 | 65 | | 57 | Post menopausal | Negative | Negative | Negative | TNBC | >5 | Positive | 3 | 3a | |

| | | | | | | | | | | | | | | |
|---|---|---|---|---|---|---|---|---|---|---|---|---|---|---|
| 57 | 180878 | 51 | Pre | | Pre menopausal | Positive | Positive | Negative | Luminal A | >5 | Negative | 2 | 3b | |
| 58 | 179082 | 50 | | 46 | Post menopausal | Negative | Negative | Negative | TNBC | >5 | Positive | 2 | 3b | |
| 59 | 191007 | 37 | Pre | | Pre menopausal | Positive | Positive | Positive | Luminal B | >5 | Positive | 2 | 3c | |
| 60 | 193507 | 61 | | 41 | Post menopausal | Positive | Positive | Negative | Luminal A | >5 | Positive | 2 | 3b | |
| 61 | 193038 | 52 | | 48 | Post menopausal | Positive | Positive | Positive | Luminal B | >5 | Positive | 2 | 3b | |
| 62 | 193806 | 65 | | 38 | Post menopausal | Positive | Positive | Negative | Luminal A | <5 | Positive | 2 | 2b | |
| 63 | 191522 | 63 | | 48 | Post menopausal | Positive | Negative | Negative | Luminal A | >5 | Positive | 1 | 3b | |
| 64 | 190233 | 47 | | 46 | Post menopausal | Positive | Positive | Negative | Luminal A | >5 | Positive | 2 | 2a | |
| 65 | 190750 | 55 | | 40 | Post menopausal | Negative | Negative | Positive | Her2+ | >5 | Negative | 2 | 3b | |
| 66 | 191488 | 59 | | 54 | Post menopausal | Negative | Negative | Negative | TNBC | <5 | Positive | 1 | 3a | |
| 67 | 189290 | 56 | Pre | | Pre menopausal | Negative | Negative | Negative | TNBC | <5 | Positive | 1 | 3a | |
| 68 | 195961 | 74 | | 48 | Post menopausal | Positive | Negative | Negative | Luminal A | <5 | Positive | 2 | 3a | |
| 69 | 167974 | 48 | Pre | | Pre menopausal | Positive | Positive | Negative | Luminal A | >5 | Positive | 4 | 3b | |
| 70 | 195165 | 58 | | 49 | Post menopausal | Positive | Positive | Positive | Luminal B | <5 | Negative | 2 | 2a | |
| 71 | 196132 | 49 | | 48 | Post menopausal | Positive | Negative | Positive | Luminal B | >5 | Positive | 2 | 3a | |
| 72 | 181695 | 35 | Pre | | Pre menopausal | Negative | Negative | Negative | TNBC | >5 | Positive | 1 | | 4 |
| 73 | 181698 | 45 | | 27 | Post menopausal | Negative | Negative | Negative | TNBC | >5 | Negative | 4 | 3b | |
| 74 | 184579 | 56 | | 41 | Post menopausal | Negative | Negative | Negative | TNBC | <5 | Positive | 3 | 2b | |
| 75 | 186996 | 48 | | 48 | Post menopausal | Positive | Negative | Negative | Luminal A | <5 | Negative | 2 | 2a | |
| 76 | 180916 | 50 | Pre | | Pre menopausal | Negative | Negative | Negative | TNBC | >5 | Positive | 1 | 2b | |
| 77 | 183745 | 32 | Pre | | Pre menopausal | Negative | Negative | Positive | Her2+ | >5 | Positive | 1 | | 4 |
| 78 | 184519 | 50 | Pre | | Pre menopausal | Negative | Negative | Negative | TNBC | >5 | Positive | 3 | 3b | |
| 79 | 189741 | 72 | | 48 | Post menopausal | Negative | Negative | Positive | Her2+ | >5 | Positive | 3 | 3a | |
| 80 | 189606 | 57 | | 47 | Post menopausal | Negative | Negative | Negative | TNBC | >5 | Negative | 3 | 3b | |
| 81 | 196140 | 63 | | 38 | Post menopausal | Positive | Positive | Positive | Luminal B | <5 | Negative | 2 | 2a | |
| 82 | 186768 | 54 | | 38 | Post menopausal | Positive | Positive | Negative | Luminal A | >5 | Negative | 3 | 3b | |
| 83 | 185226 | 52 | | 42 | Post menopausal | Positive | Negative | Negative | Luminal A | >5 | Positive | 3 | 3b | |
| 84 | 196858 | 70 | | 45 | Post menopausal | Negative | Negative | Positive | Her2+ | <5 | Negative | 2 | 2a | |
| 85 | 196334 | 36 | Pre | | Pre menopausal | Positive | Negative | Negative | Luminal A | <5 | Negative | 3 | 2a | |
| 86 | 197424 | 44 | | 43 | Post menopausal | Negative | Negative | Negative | TNBC | >5 | Positive | 2 | 3b | |

| | | | | | | | | | | | | | | |
|---|---|---|---|---|---|---|---|---|---|---|---|---|---|---|
| 87 | 189790 | 43 | Pre | | Pre menopausal | Negative | Negative | Negative | TNBC | >5 | Negative | 3 | 2b | |
| 88 | 197432 | 42 | Pre | | Pre menopausal | Positive | Positive | Negative | Luminal A | <5 | Negative | 2 | 2a | |
| 89 | 190574 | 40 | Pre | | Pre menopausal | Negative | Negative | Negative | TNBC | >5 | Positive | 2 | 3b | |
| 90 | 186390 | 65 | | 60 | Post menopausal | Negative | Negative | Positive | Her2+ | <5 | Positive | 3 | 3c | |
| 91 | 196204 | 64 | | 44 | Post menopausal | Negative | Negative | Positive | Her2+ | >5 | Positive | 3 | 3b | |
| 92 | 188005 | 37 | Pre | | Pre menopausal | Negative | Negative | Negative | TNBC | <5 | Positive | 2 | 2b | |
| 93 | 193295 | 29 | Pre | | Pre menopausal | Negative | Negative | Negative | TNBC | >5 | Positive | 4 | 3b | |
| 94 | 149395 | 40 | Pre | | Pre menopausal | Negative | Negative | Negative | TNBC | <5 | Positive | 2 | 3b | |
| 95 | 195486 | 32 | Pre | | Pre menopausal | Positive | Positive | Positive | Luminal B | <5 | Positive | 2 | 3c | |
| 96 | 188594 | 27 | Pre | | Pre menopausal | Negative | Negative | Negative | TNBC | <5 | Negative | 4 | 1a | |
| 97 | 200180 | 52 | | 44 | Post menopausal | Negative | Negative | Positive | Her2+ | <5 | Negative | 4 | 2a | |
| 98 | 200190 | 45 | | 44 | Post menopausal | Positive | Positive | Positive | Luminal B | >5 | Positive | 2 | 3c | |
| 99 | 200728 | 60 | | 44 | Post menopausal | Positive | Positive | Negative | Luminal A | >5 | Positive | 2 | 3a | |
| 100 | 193046 | 46 | | 44 | Post menopausal | Negative | Negative | Negative | TNBC | >5 | Positive | 4 | | 4 |
| 101 | 264320 | 36 | Pre | | Pre menopausal | Negative | Negative | Positive | Her2+ | >5 | Positive | 3 | 3b | |
| 102 | 263916 | 65 | | 36 | Post menopausal | Negative | Negative | Positive | Her2+ | >5 | Positive | 3 | 3a | |
| 103 | 263225 | 57 | | 48 | Post menopausal | Negative | Negative | Negative | TNBC | <5 | Positive | 4 | 2b | |
| 104 | 263107 | 32 | Pre | | Pre menopausal | Positive | Positive | Negative | Luminal A | <5 | Positive | 2 | 2a | |
| 105 | 271833 | 74 | | 48 | Post menopausal | Negative | Negative | Negative | TNBC | <5 | Positive | 2 | 2b | |
| 106 | 260604 | 60 | | 40 | Post menopausal | Negative | Negative | Negative | TNBC | >5 | Positive | 2 | 3b | |
| 107 | 264585 | 66 | | 46 | Post menopausal | Negative | Negative | Negative | TNBC | >5 | Positive | 3 | 3b | |
| 108 | 263299 | 60 | | 54 | Post menopausal | Positive | Positive | Positive | TNBC | >5 | Positive | 3 | 3b | |
| 109 | 257740 | 56 | | 45 | Post menopausal | Negative | Negative | Positive | Her2+ | >5 | Positive | 3 | 3b | |
| 110 | 271925 | 73 | | 53 | Post menopausal | Positive | Positive | Negative | Her2+ | <5 | Positive | 3 | 3a | |
| 111 | 260885 | 46 | | 35 | Post menopausal | Negative | Negative | Negative | TNBC | >5 | Positive | 3 | 3b | |
| 112 | 272954 | 35 | Pre | | Pre menopausal | Positive | Positive | Negative | Luminal A | >5 | Positive | 2 | 3b | |
| 113 | 265506 | 38 | Pre | | Pre menopausal | Negative | Negative | Negative | TNBC | >5 | Positive | 2 | 3b | |
| 114 | 264835 | 62 | Pre | | Pre menopausal | Negative | Negative | Negative | TNBC | >5 | Positive | 3 | 3b | |
| 115 | 240488 | 50 | | 45 | Post menopausal | Negative | Negative | Negative | TNBC | >5 | Positive | 2 | | 4 |
| 116 | 235299 | 36 | Pre | | Pre menopausal | Negative | Negative | Negative | TNBC | >5 | Positive | 3 | 3a | |

| | | | | | | | | | | | | | | |
|---|---|---|---|---|---|---|---|---|---|---|---|---|---|---|
| 117 | 265181 | 56 | | 52 | Post menopausal | Positive | Negative | Negative | Luminal A | >5 | Negative | 2 | 3b | |
| 118 | 265419 | 35 | Pre | | Pre menopausal | Negative | Negative | Negative | TNBC | >5 | Negative | 3 | 3b | |
| 119 | 262684 | 36 | Pre | | Pre menopausal | Negative | Negative | Negative | TNBC | >5 | Positive | 3 | 3b | |
| 120 | 263916 | 65 | | 36 | Post menopausal | Negative | Negative | Positive | Her2+ | >5 | Positive | 3 | 3a | |
| 121 | 203377 | 77 | | 48 | Post menopausal | Negative | Negative | Negative | TNBC | >5 | Positive | 3 | | 4 |
| 122 | 271932 | 68 | | 40 | Post menopausal | Positive | Positive | Negative | Luminal A | <5 | Negative | 3 | 2a | |
| 123 | 270760 | 54 | | 49 | Post menopausal | Positive | Negative | Negative | Luminal A | >5 | Positive | 2 | 3b | |
| 124 | 257444 | 40 | | 40 | Post menopausal | Negative | Negative | Negative | TNBC | >5 | Positive | 4 | 3b | |
| 125 | 264426 | 26 | | 24 | Post menopausal | Positive | Positive | Negative | Luminal A | >5 | Positive | 2 | 3c | |
| 126 | 259421 | 56 | | 49 | Post menopausal | Negative | Negative | Negative | TNBC | >5 | Positive | 2 | 3a | |
| 127 | 273820 | 32 | Pre | | Pre menopausal | Positive | Positive | Negative | Luminal A | >5 | Positive | 2 | 3a | |
| 128 | 276409 | 67 | | 42 | Post menopausal | Positive | Positive | Negative | Luminal A | >5 | Positive | 2 | 3a | |
| 129 | 278287 | 58 | | 40 | Post menopausal | Positive | Positive | Negative | Luminal A | <5 | Positive | 3 | 2b | |
| 130 | 270337 | 47 | | 45 | Post menopausal | Negative | Negative | Negative | TNBC | >5 | Positive | 3 | 3c | |
| 131 | 267654 | 35 | Pre | | Pre menopausal | Positive | Negative | Negative | Luminal A | >5 | Negative | 2 | 3b | |
| 132 | 259651 | 52 | | 34 | Post menopausal | Negative | Negative | Negative | TNBC | <5 | Negative | 2 | 2a | |
| 133 | 271271 | 63 | | 44 | Post menopausal | Negative | Negative | Negative | TNBC | >5 | Positive | 3 | 3b | |
| 134 | 273688 | 38 | Pre | | Pre menopausal | Positive | Negative | Positive | Luminal B | <5 | Negative | 4 | | 4 |
| 135 | 213554 | 32 | Pre | | Pre menopausal | Positive | Positive | Positive | Luminal B | >5 | Positive | 3 | 3b | |
| 136 | 269059 | 27 | Pre | | Pre menopausal | Positive | Positive | Negative | Luminal A | >5 | Positive | 2 | 3b | |
| 137 | 267923 | 70 | | 52 | Post menopausal | Negative | Negative | Positive | Her2+ | >5 | Positive | 2 | 3b | |
| 138 | 263385 | 44 | | 42 | Post menopausal | Positive | Positive | Negative | Luminal A | >5 | Positive | 3 | | 4 |
| 139 | 264320 | 36 | Pre | | Pre menopausal | Negative | Negative | Positive | Her2+ | >5 | Positive | 3 | 3b | |
| 140 | 264481 | 42 | Pre | | Pre menopausal | Negative | Negative | Positive | Her2+ | <5 | Negative | 3 | | 1 |
| 141 | 249497 | 25 | Pre | | Pre menopausal | Negative | Negative | Positive | Her2+ | <5 | Negative | 3 | 2a | |
| 142 | 246624 | 35 | Pre | | Pre menopausal | Positive | Positive | Positive | Luminal B | >5 | Negative | 2 | 2b | |
