# Supplementary material for: Evaluating the role of MEN1 gene expression and its clinical significance in breast cancer patients
Source: PLoS One. 2023 Jul 12;18(7):e0288482. doi: 10.1371/journal.pone.0288482 (PMC10337982; doi:10.1371/journal.pone.0288482)
Supplement: S1 File — (DOCX) [file pone.0288482.s002.docx]

**Uncropped images and blots incorporated in the Manuscript**

A


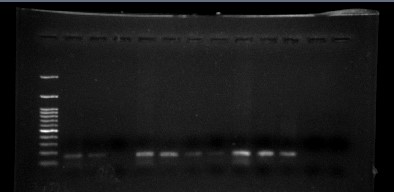


B


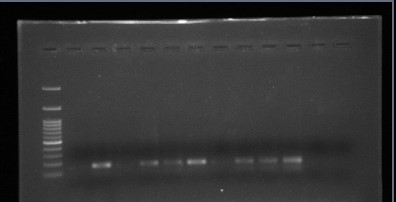


Figure 3: (A) Representative Agarose gel picture for MS-PCR showing promoter methylation (product size: M = 161) and unmethylation status (product size: UM = 160) of MEN1 in tumor tissue and adjacent normal breast tissue. (B) MS-PCR results exhibiting the MEN1 promoter methylation and unmethylation status of breast tumor tissue.

A


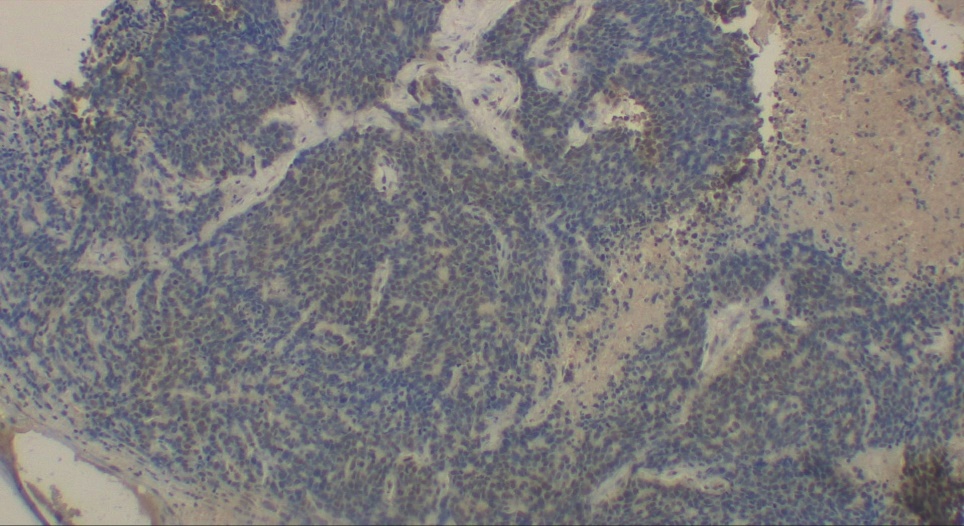


B


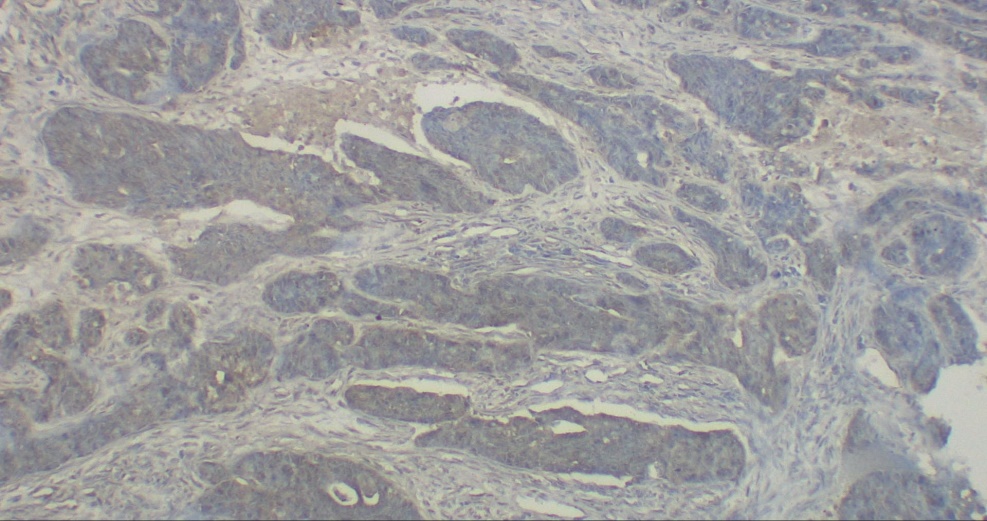


C


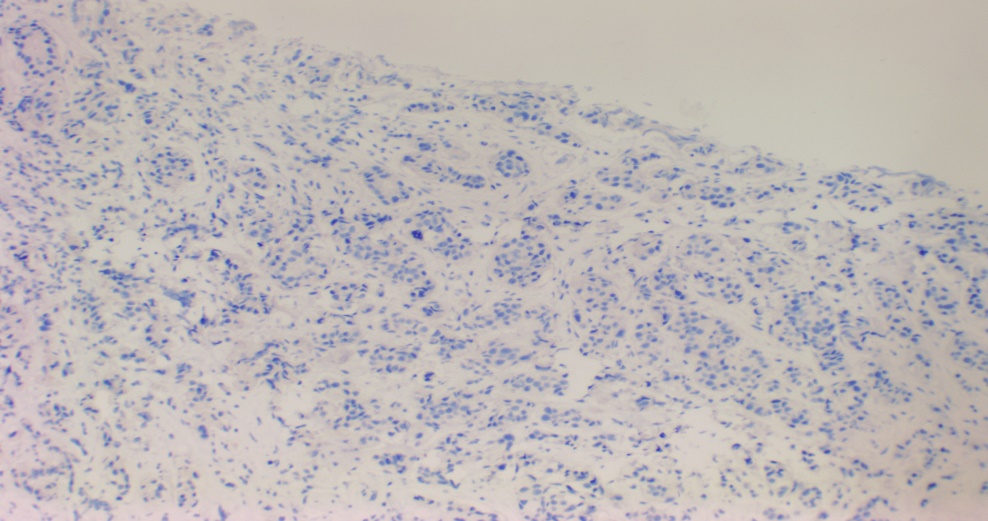


Figure 4: Representative panel of immunohistochemical images taken at 20X magnification for MEN1 protein detection in breast tissue (A) Normal Breast tissue (B) Breast cancer tissue showing low expression of menin protein (C) Breast cancer tissue with high expression of menin protein.


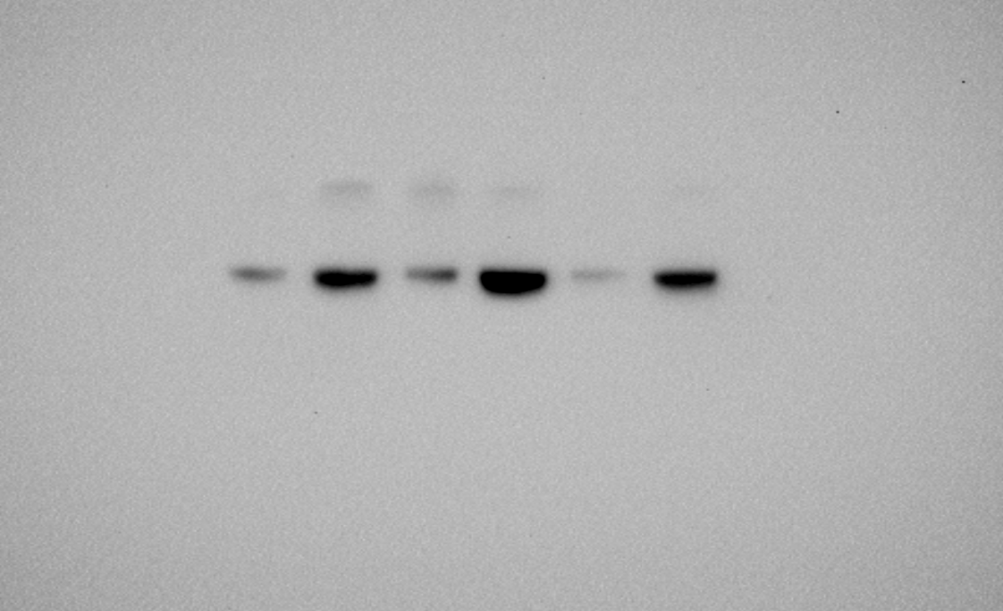


Uncropped menin blot


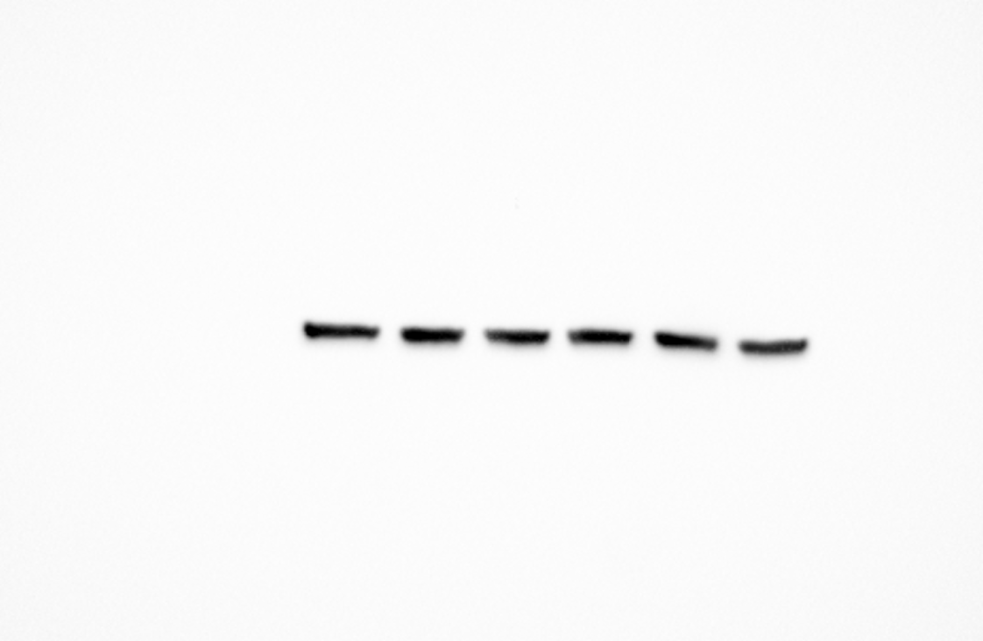


Uncropped beta actin blot

Figure 5: MEN1 protein expression analysis through western blot (A) elevated expression of menin in tumor tissue as compared to its paired normal breast tissue

A


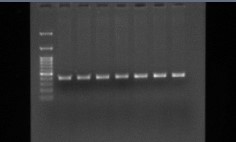


B


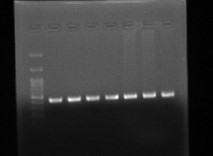


C


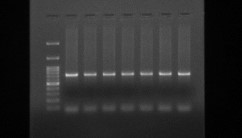


Figure 6: Representative agarose gel images showing PCR amplification and electrophoregrams of MEN1 exon 8, 9 and 10 for mutation analysis.
