# Supplementary figures and images for: Evaluating the role of MEN1 gene expression and its clinical significance in breast cancer patients
Source: PLoS One. 2023 Jul 12;18(7):e0288482. doi: 10.1371/journal.pone.0288482 (PMC10337982; doi:10.1371/journal.pone.0288482)

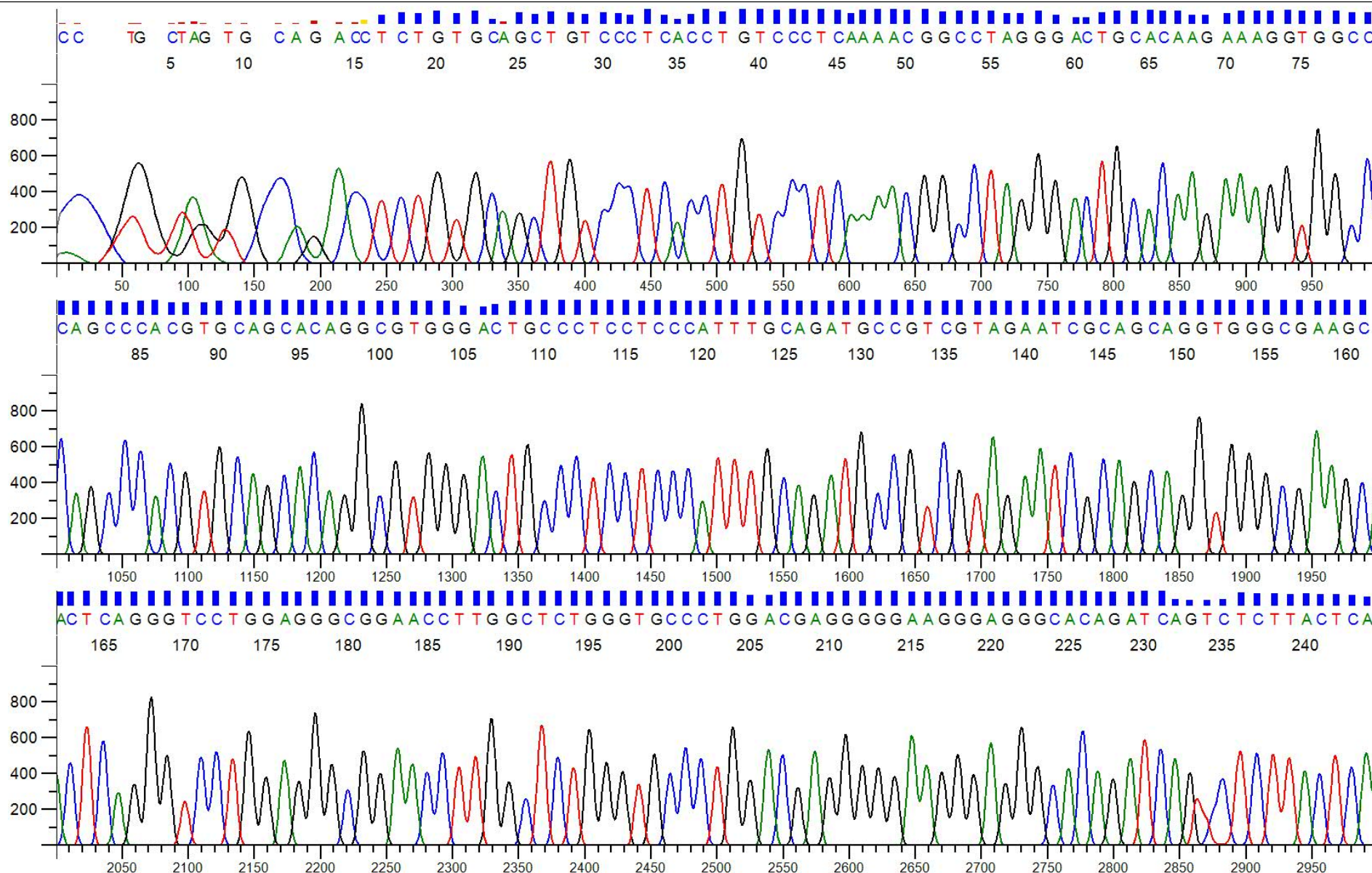

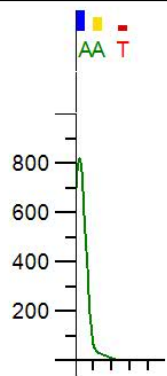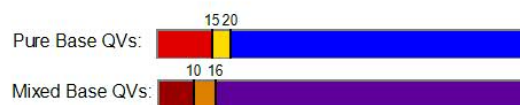

Supplement: S2 File — (ZIP) [file pone.0288482.s003.zip › ME9.pdf]

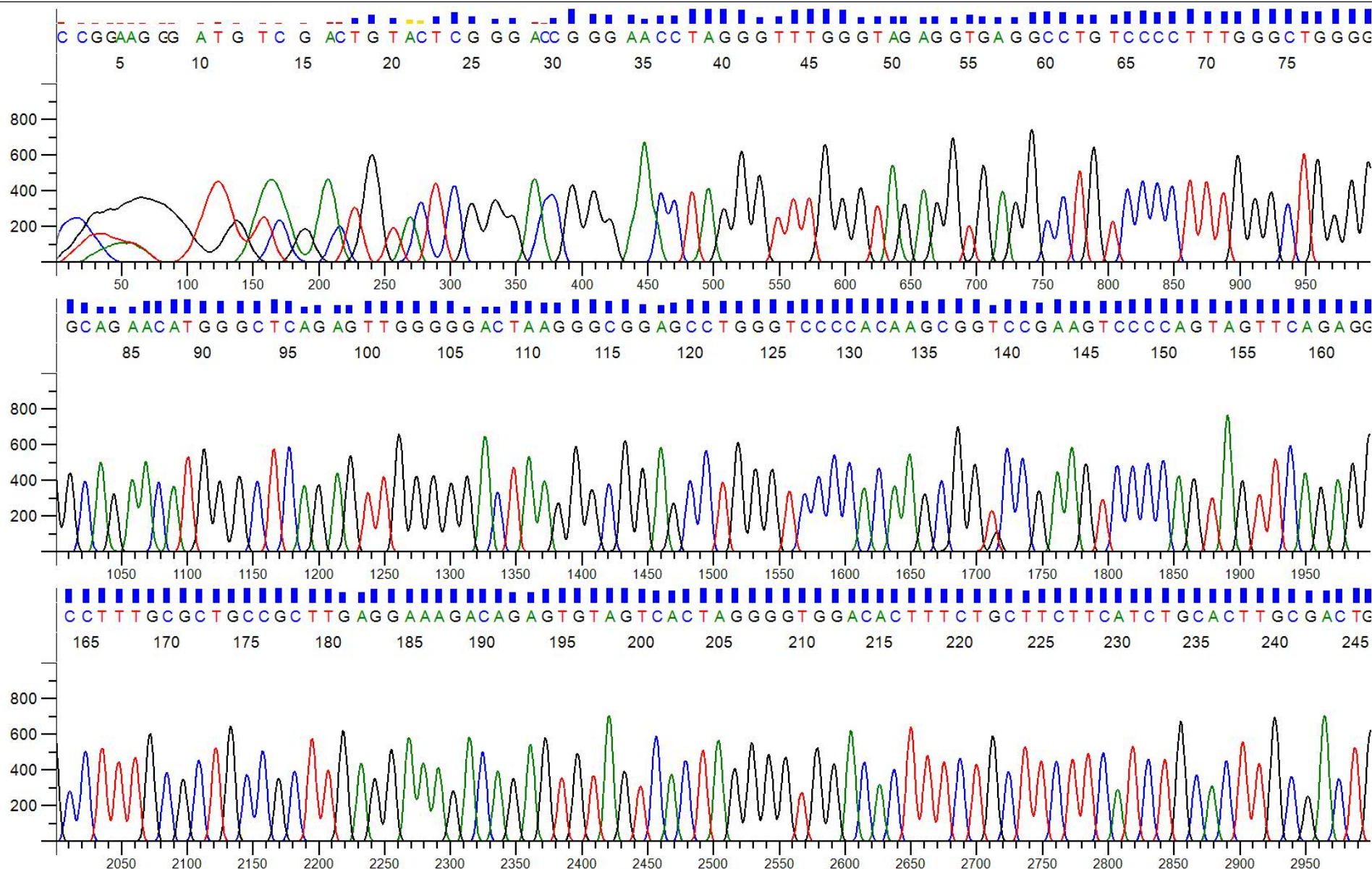

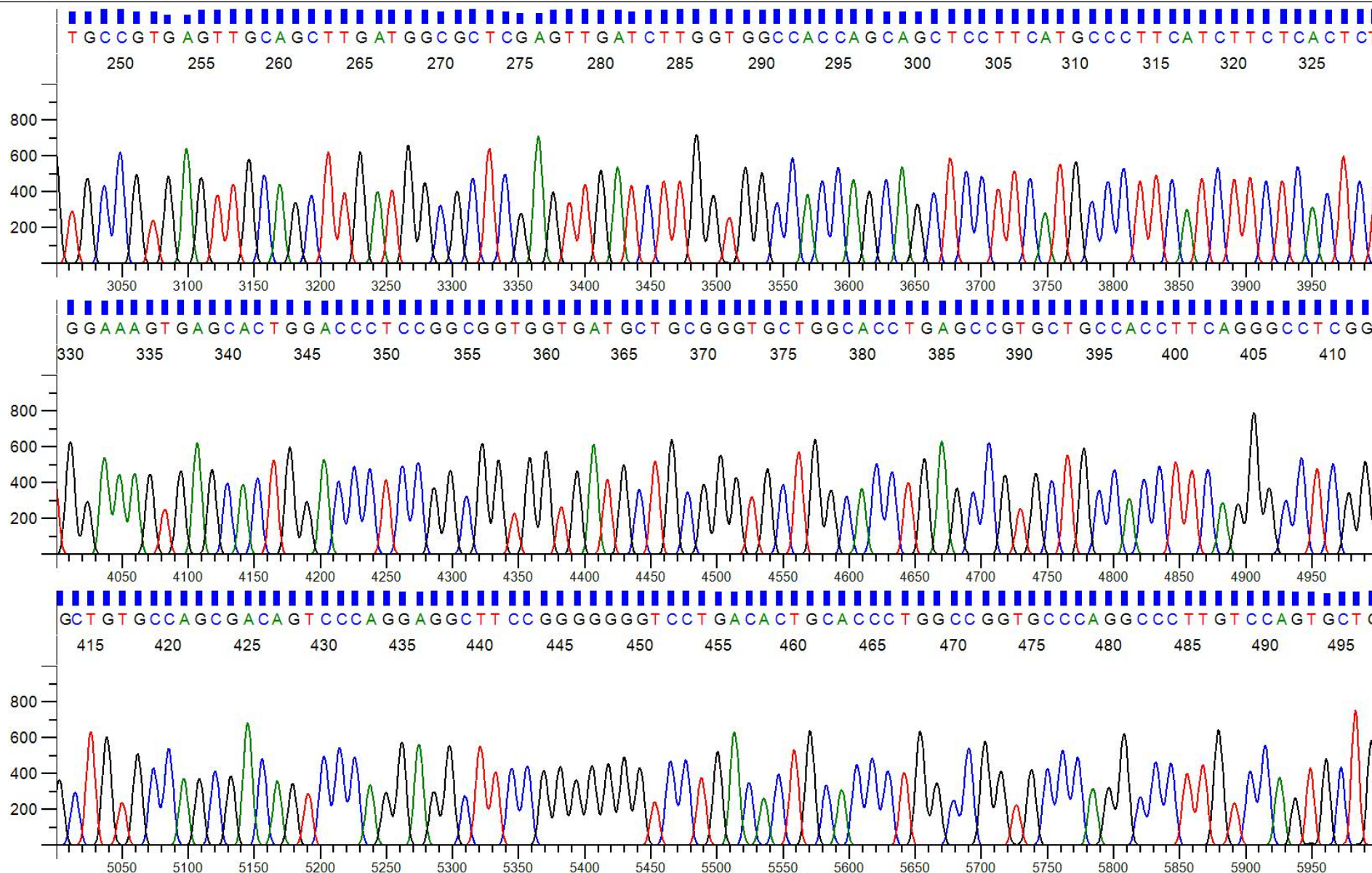

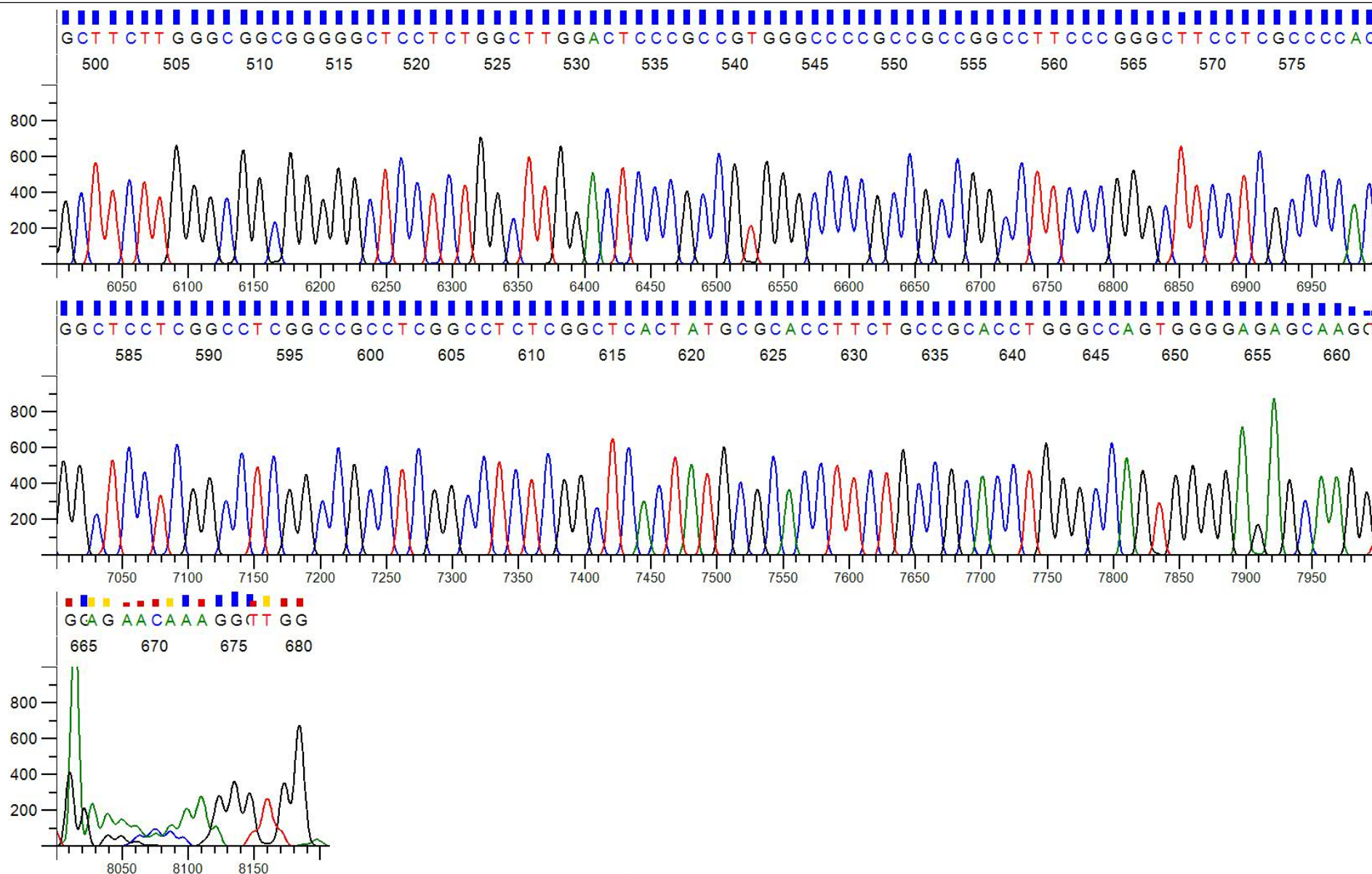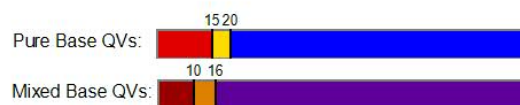

Supplement: S2 File — (ZIP) [file pone.0288482.s003.zip › ME10.pdf]

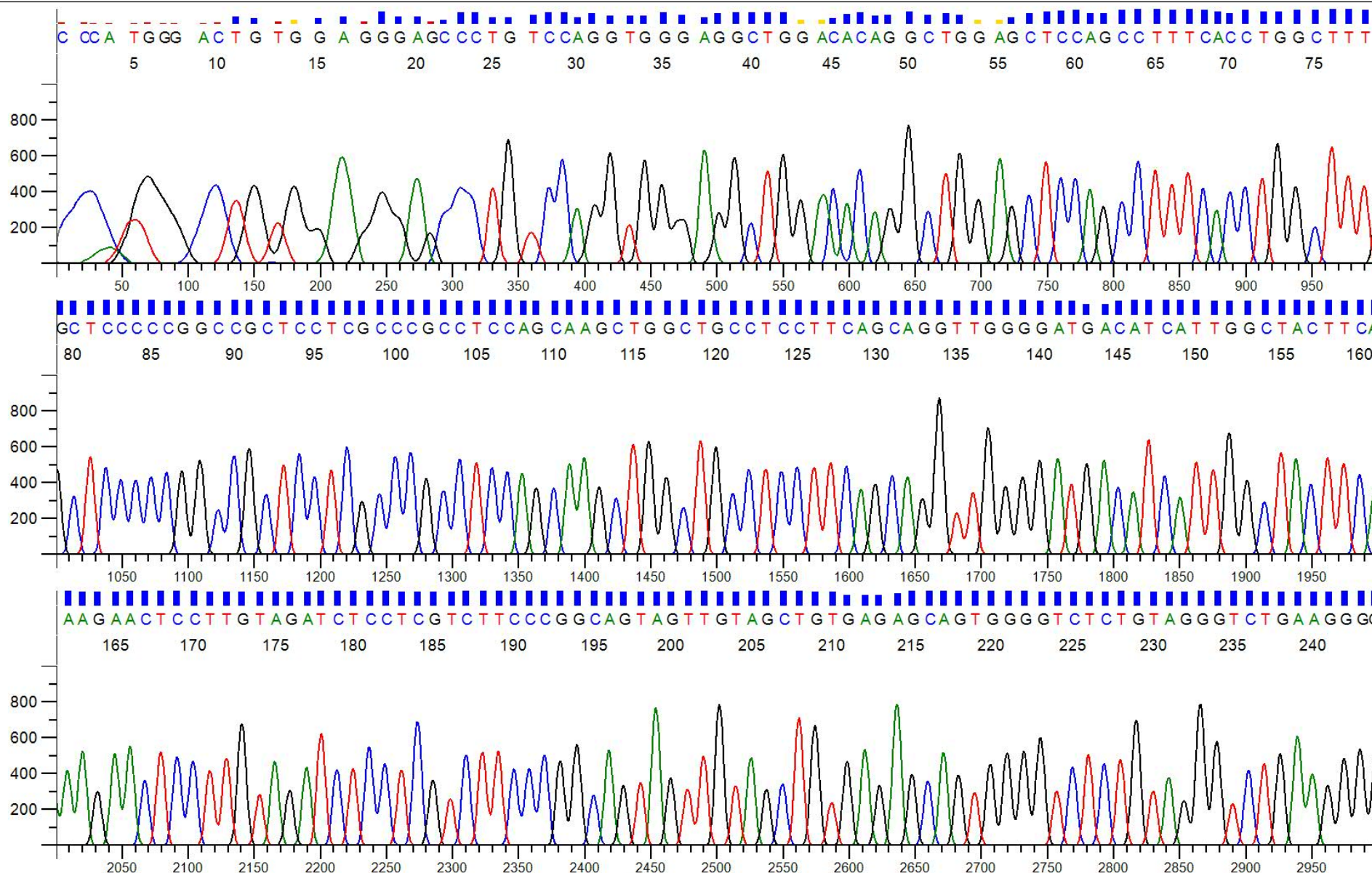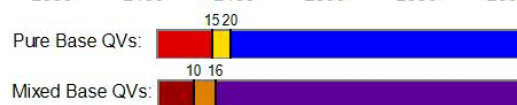

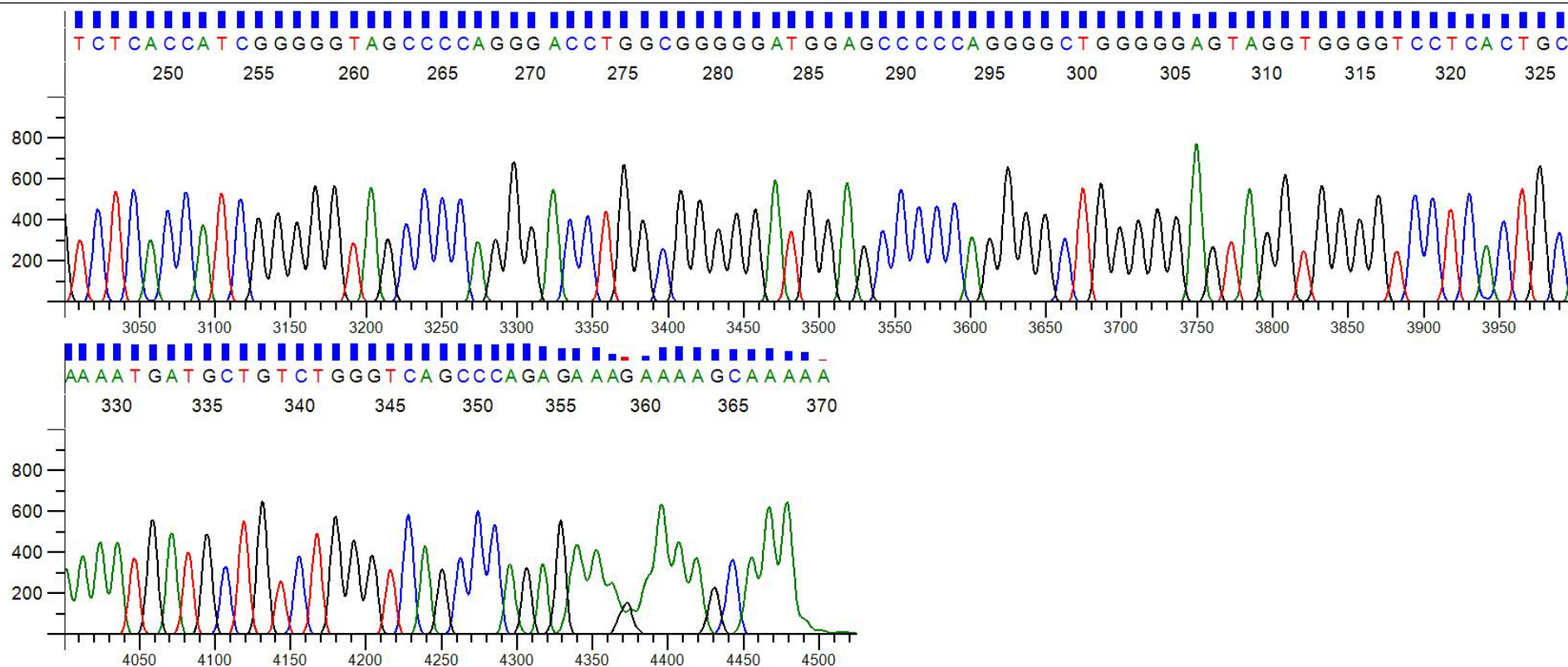

Supplement: S2 File — (ZIP) [file pone.0288482.s003.zip › ME8.pdf]
